# Antigenicity of the 2015–2016 seasonal H1N1 human influenza virus HA and NA proteins

**DOI:** 10.1371/journal.pone.0188267

**Published:** 2017-11-16

**Authors:** Amelia M. Clark, Marta L. DeDiego, Christopher S. Anderson, Jiong Wang, Hongmei Yang, Aitor Nogales, Luis Martinez-Sobrido, Martin S. Zand, Mark Y. Sangster, David J. Topham

**Affiliations:** 1 David H. Smith Center for Vaccine Biology and Immunology, University of Rochester Medical Center, Rochester, New York, United States of America; 2 Division of Nephrology, Department of Medicine, University of Rochester Medical Center, Rochester, New York, United States of America; 3 Department of Biostatistics and Computational Biology, University of Rochester Medical Center, Rochester, New York, United States of America; 4 Department of Microbiology and Immunology, University of Rochester Medical Center, Rochester, New York, United States of America; University of Georgia, UNITED STATES

## Abstract

Antigenic drift of the hemagglutinin (HA) and neuraminidase (NA) influenza virus proteins contributes to reduced vaccine efficacy. To analyze antigenic drift in human seasonal H1N1 viruses derived from the 2009 pandemic H1N1 virus (pH1N1-like viruses) accounts for the limited effectiveness (around 40%) of vaccination against pH1N1-like viruses during the 2015–2016 season, nasal washes/swabs collected from adult subjects in the Rochester, NY area, were used to sequence and isolate the circulating viruses. The HA and NA proteins from viruses circulating during the 2015–2016 season encoded eighteen and fourteen amino acid differences, respectively, when compared to A/California/04/2009, a strain circulating at the origin of the 2009 pandemic. The circulating strains belonged to subclade 6B.1, defined by HA amino acid substitutions S101N, S179N, and I233T. Hemagglutination-inhibiting (HAI) and HA-specific neutralizing serum antibody (Ab) titers from around 50% of pH1N1-like virus-infected subjects and immune ferrets were 2–4 fold lower for the 2015–2016 circulating strains compared to the vaccine strain. In addition, using a luminex-based mPlex HA assay, the binding of human sera from subjects infected with pH1N1-like viruses to the HA proteins from circulating and vaccine strains was not identical, strongly suggesting antigenic differences in the HA protein. Additionally, NA inhibition (NAI) Ab titers in human sera from pH1N1-like virus-infected subjects increased after the infection and there were measurable antigenic differences between the NA protein of circulating strains and the vaccine strain using both ferret and human antisera. Despite having been vaccinated, infected subjects exhibited low HAI Ab titers against the vaccine and circulating strains. This suggests that poor responses to the H1N1 component of the vaccine as well as antigenic differences in the HA and NA proteins of currently circulating pH1N1-like viruses could be contributing to risk of infection even after vaccination.

## Introduction

Circulating influenza viruses are estimated by the World Health Organization (WHO) to infect 5–10% of the population every year, causing a morbidity rate of 3 to 5 million and a mortality rate of 250,000–500,000 deaths annually (www.who.int/mediacentre/factsheets/fs211/en/). Influenza A (IAV) and B (IBV) viruses are members of the family *Orthomyxoviridae*, whose entire genome is encoded by 8 single-stranded, negative-sense RNA segments [1]. IAVs are classified by subtype according to the antigenic reactivity and sequences of hemagglutinin (HA) and neuraminidase (NA), the two major virion surface glycoproteins.

The quaternary structure of the influenza HA protein is a homotrimer, which is “primed” for activation at low pH by proteolytic cleavage of the trimeric HA0 precursor to form two subunits (HA1 and HA2), enabling virus entry into cells and HA-mediated fusion activity [2]. HA1 forms a globular head domain at the membrane-distal tip of the HA protein [3]. At least five distinct antigenic sites have been described in the globular head domain of the protein (designated Sa, Sb, Ca1, Ca2, and Cb, for H1N1 viruses) [4]. The HA protein interacts with *N*-acetylneuraminic (sialic) acid residues of host cell glycoconjugates via the receptor-binding site (RBS), located atop the HA head [5]. The RBS is characterized by four elements: the 190-alpha helix, the 130-loop, the 220-loop, and a hydrogen-bonded network of conserved amino acids at positions 98, 153, 183, and 195 (H3 numbering) [5]. HA2 together with part of HA1 form the stalk domain, which includes the transmembrane domain, the fusion peptide, and the cytoplasmic tail [6,7].

Since 2009, the HA genes from pH1N1 viruses have evolved. For well over a year, viruses in clade 6, and carrying amino acid substitutions of D114N, S202T (in antigenic site Sb), and S220T (in antigenic site Ca1) in the head domain, and E391K and S468N in the stalk domain compared with A/Cal/07/09, have predominated worldwide, with a number of subclades emerging. Most of the viruses characterized since September 2014 carry HA genes in subclade 6B, which is characterized by additional amino acid substitutions of K180Q (in antigenic site Sa), A273T and K300E in the head domain and E516K in the stalk domain compared with A/Cal/07/09. A number of virus clusters have emerged within clade 6B and two of these have been designated as subclades: viruses in subclade 6B.1 are defined by head amino acid substitutions S101N, S179N (in antigenic site Sa) and I233T, while those in subclade 6B.2 are defined by head amino acid substitutions V169T (in antigenic site Sa) and V190I [8,9].

The NA protein is a receptor-destroying enzyme that functions as a sialidase, facilitating the release of progeny virions by catalyzing the cleavage of terminal sialic acid residues from cellular and viral proteins [10]. In addition, data suggests that NA protein allows influenza to penetrate the mucus layer secreted in the host respiratory epithelia by cleaving the sialydated glycoproteins present in the mucus [11]. The NA protein quaternary structure is a homotetramer in which each monomeric subunit forms a stalk domain and a head domain, the structure of which is analogous to a six-bladed propeller and upon which is located the active site [12].

Humans are most commonly infected with IAV subtypes H3N2 or H1N1, or one of two IBV lineages. Accordingly, the seasonal influenza vaccines include 3 or 4 viral strains (H1N1, H3N2, and one or two influenza B viruses) [13]. Antibodies directed against the head of the HA, blocking HA attachment to sialic acid-bearing proteins on the cell surface, are the most abundant of the neutralizing antibodies elicited after vaccination and natural viral infection [14]. However, protective HA stalk- and NA-specific neutralizing antibodies, which respectively inhibit membrane fusion [15] and NA-catalyzed release of progeny virions from cells [16], have been found in humans [16–20].

The HA and NA proteins incorporate mutations through a process called antigenic drift, decreasing vaccine efficacy [21,22], sometimes by incorporating as little as one point mutation [23]. This has led to the need for yearly vaccinations in an effort to protect the general populace from infection. Despite regularly updating the vaccine strains to better reflect circulating strains, vaccination does not protect 100% against infection [24]. It is routine for the WHO and the Centers for Disease Control and Prevention (CDC) to conduct vaccine efficacy studies and surveil circulating strains for antigenic variants. Currently circulating seasonal H1N1 viruses are related to the pandemic H1N1 virus that emerged in 2009 (pH1N1-like) [8,25]. Interestingly, even after 7 years without an update to the vaccine strain and the emergence of several genetic subgroups, recently circulating H1N1 viruses (the majority of which fall into subclade 6B.1, defined by amino acid substitutions S101N, S179N, and I233T [8], have still been considered antigenically similar to the A/California/07/2009 (A/Cal/07/09), an early isolate of the pandemic. This is due to the absence of significant antigenic differences measured in the HA head by Hemagglutination-inhibiting HAI assays using sera from ferrets immunized with A/Cal/07/09 [8,25]. However, most studies of antigenic drift are done at the population level, with few studies of the immune state at the time of infection and antigenicity of the infecting virus from the same individual. Additionally, antigenic differences among influenza viruses are traditionally evaluated by differences between HA proteins, with studies rarely taking into account antigenic differences between the viral NA proteins.

Despite the apparent antigenic similarity of the vaccine strain and circulating H1N1 strains, a study conducted by the United States Flu Vaccine Effectiveness Network found that 41% of the 781 participants infected with an pH1N1-like IAV during the 2015–16 season had previously been vaccinated (https://www.cdc.gov/flu/about/season/flu-season-2015-2016.htm). Moreover, previous work from our group showed that people infected with 2010–2011 and 2012–2013 seasonal H3N2 viruses uniformly had lower Ab titers against the virus they were infected with compared to the strain used for vaccination that season, suggesting that antigenic differences from the vaccine contributed to increased risk of infection [26]. We sought to determine whether the same was true for human seasonal pH1N1-like viruses. To this end, we collected nasal washes/swabs as well as serum at the time of illness presentation (day 0) and in convalescence (day 28) from infected adults in the Rochester, NY area. The viruses infecting each individual patient were isolated from nasal washes/swabs, and analyzed for sequence-based and antigenic differences. The HA of the pH1N1-like viruses showed small but measurable antigenic differences to the vaccine strain using ferret and human sera in HAI, microneutralization (MN) and m-plex HA assays. Additionally, infected subjects exhibited low HAI Ab titers against the vaccine strain, despite being vaccinated, and the titers increased after infection, in the three assays. In addition, we did measure antigenic differences between the NA of the pH1N1-like viruses and the vaccine strain. This suggests that poor responses to the H1N1 component of the vaccine, and differences in antigenicity in the HA and NA proteins of the circulating pH1N1-like viruses could be contributing to risk of infection even after vaccination. Moreover, the mPlex-Flu HA assay suggests immune adaptation to the infecting virus after pH1N1 infection (day 28 sera) and Original Antigenic Sin (OAS)-type responses in most of the subjects using the sera collected during the illness visit (day 0).

## Materials and methods

### Study design and human subjects

Human patients were enrolled as part of an acute influenza surveillance protocol, in which patients presenting influenza-like symptoms were asked to visit the University of Rochester Vaccine Research Unit for sampling by nasal wash and nasopharyngeal swab (combined, nasal wash/swab). Patients were named with a number and the acronym ACU (from Acute influenza). Sera from patients enrolled in the acute influenza surveillance protocol were obtained at the acute visit (day 0), and around 28 days after the acute visit (day 28). Only samples from patients whom tested positive via RT-PCR for IAV H1N1 were included in this study. Some patients had received the 2014–15 or 2015–16 flu vaccine prior to infection (S1 Table). Each study was approved by the University of Rochester Human Research Subjects Review Board (protocol 14–0101). Informed written consent was obtained for each participant.

### Cells and viruses

Madin-Darby canine kidney (MDCK) cells and human embryonic kidney 293T cells were obtained from the American Type Culture Collection (ATCC, CCL-34 and CRL-11268, rspectively). MDCK cells constitutively expressing the HA protein of A/Indonesia/5/2005 H5N1 (MDCK/H5) were previously described [27]. MDCK and MDCK/H5 cells were grown at 37°C in air enriched with 5% CO_2_, in Dulbecco’s modified minimal essential medium (DMEM) (Corning, Manassas, VA; #10-013-CV) supplemented with 10% (v/v) fetal bovine serum (FBS) (Lonza, Walkersville, MD; #14-502F) and 10 μg/mL gentamycin (Gibco, Grand Island, NY; #15710–064). MDCK/H5 cells were grown in media additionally supplemented with 200 μg/mL hygromycin B every other passage to ensure continued expression of H5, as previously described [27,28].

A virus stock of influenza virus A/California/04/2009 (A/Cal/04/09), produced in 9 to 11-day-old SPF embryonated chicken eggs, incubated 2 to 3 days at 33.5 C, was used as the reference strain for HAI and MN assays (BEI Resources, Manassas, VA; NR-13659).

### Virus isolation and titrations

MDCK cells were inoculated with nasal washes/swabs from pH1N1-like virus-infected patients. Upon observation of cytopathic effect, supernatants were collected and used to infect fresh cells. If no cytopathic effect was observed, supernatants were collected 72 hours post-infection (hpi) and used to infect fresh cells. A maximum of three serial passages was performed. All infected cells were grown at 37°C in air enriched with 5% CO_2_, in DMEM supplemented with 0.3% bovine serum albumin (BSA) (Gibco; #15260–037), 10 μg/mL gentamycin, and 1 μg/mL tosylsulfonyl phenylalanyl chloromethyl ketone (TPCK)-treated trypsin (Worthington Biochemical, Lakewood, NJ; #3570).

Viruses were titrated by immunofocus assay using an IAV monoclonal Ab specific for the viral NP protein (HB-65), as previously described [29,30]. IAV NP-positive cells were visualized and enumerated to determine virus titers (fluorescent-forming units, FFU/ml) using a fluorescence microscope.

### HA and NA sequencing

Viral RNA was purified from nasal swabs/washes using the QIAamp viral RNA extraction kit (Qiagen, Hilden, Germany) or from cell culture extracts using the RNeasy mini kit (Qiagen) according to the manufacturer’s instructions. Reverse transcription was performed to obtain cDNA of the HA or NA genes using primers H1N1-HA-5’NCR-VS (5’-GGGGAAAACAAAAGCAACAAAAATG-3’) and H1N1-HA-3’-NCR-RS (GTGTTTTTCTCATGCTTCTGAAATCCTAATG-3’) or NA-A/Cal-1-VS (5’-ATGAATCCAAACCAAAAGATAATAACCATT-3’) and NA-A/Cal-1410-RS (5’-TTACTTGTCAATGGTAAATGGCAACTC-3’), respectively, with the High Capacity cDNA reverse transcription kit (Applied Biosystems; #4368814).

To obtain DNA segments encoding the HA or NA genes to sequence the HA and NA genes, overlapping PCR using primers H1N1-HA-5’NCR-VS and H1N1-HA-923-RS (GGGAGGCTGGTGTTTATAGCACCC-3’) (for HA region 1) and H1N1-HA-793-VS (CTAGTGGTACCGAGATATGCATTCGC-3’) and H1N1-HA-3’-NCR-RS (for HA region 2) or NA-A/Cal-1-VS and NA-A/Cal-755-RS (5’-GAGGCCTGTCCATTACTTGGTCC-3’) (for NA region 1), and NA-A/Cal-652-VS (5’-GAGTTGGAGAAACAATATATTGAGAACACAAG-3’) and NA-A/Cal-1410-RS (for NA region 2), respectively, was performed with the Platinum Pfx polymerase (Life Technologies; #11708021). The VS and RS primers used in each PCR were used for Sanger sequencing. The complete HA and NA sequences were obtained directly amplifying the viral RNA from nine of the nasal swabs/washes. In addition, viruses from 9 of the 14 clinical samples were isolated, and the complete HA (GenBank: KY056604, KY056601, KY056612, KY056603, KY056600, KY056608, KY056614, KY056613, KY056606) and NA (GenBank: KY056597, KY056599, KY056602, KY056609, KY056598, KY056607, KY056605, KY056610, KY056611) sequences were obtained from the viruses isolated in MDCK cells.

### Plasmids

Ambisense pDZ plasmids [31] encoding the NA genes of A/Cal/04/09 (NA-wt) or the virus isolated from patient 001 (NA-001) were obtained by overlapping PCR. Viral RNA purified from the nasal swab/wash purified from patient 001 (NA-001) or cells infected with A/Cal/04/09 (BEI Resources, NR-13659) (NA-wt) was reverse transcribed using primers NCR-SapI-NACal-5-VS (5’-GATCGCTCTTCTGGGAGCAAAAGCAGGAGTTTAAAATGAATCCAAACCAAAAG-3’) and NCR-SapI-NACal-3-RS (5’-CATCGCTCTTCTATTAGTAGAAACAAGGAGTTTTTTGAACAAATTACTTGTCAATGGT-3’) and the High Capacity cDNA reverse transcription kit (Applied Biosystems) to produce cDNA. These cDNAs were then amplified by PCR using the same primers NCR-SapI-NACal-5-VS and NCR-SapI-NACal-3-RS and the Platinum Pfx polymerase (Life Technologies) to obtain the DNA transcript of the NA gene with SapI restriction sites at the 5’ and 3’ ends. PCR products were digested with the SapI restriction enzyme and cloned into the pDZ plasmid.

### Virus rescues

H5-pseudotyped single cycle infectious IAVs (sciIAVs) in which the viral HA gene was replaced by a gene encoding green fluorescent protein (GFP) and the viral NA gene was replaced either by the NA encoded by A/Cal/04/09 (NA-wt) or by the virus isolated from patient 001 (NA-001) were generated in the backbone of A/Cal/04/09 as previously described [27,32]. Briefly 293T cells and MDCK cells stably expressing the HA protein from the H5N1 strain A/Indonesia/5/2005 (MDCK/H5) [27], complementing the absence of the HA gene in the sciIAV genome, were co-cultured at a 1:1 ratio in 6-well plates were transiently co-transfected, in suspension, with 1 μg each of ambisense plasmids encoding the six A/Cal/04/09 genes (excluding HA and NA) (pDZ-PB2, -PB1, -PA, -NP, -NS, -M, and -GFP) (kindly provided by A. Garcia-Sastre, Icahn School of Medicine at Mount Sinai, NY) and GFP plus the pDZ-NAs encoding NA-wt or NA-001 proteins, using DNA-IN (Molecular Transfec, Inc; #73771) [27,28]. Stocks were titrated by immunofocus assay (FFU/ml) on MDCK cells as described above. The identity of the NA ORFs in the rescued viruses was confirmed by restriction digestion and sequencing (Genewiz). Before using the sciIAVs in assays, it was confirmed that neither the ferret nor human sera samples contained antibodies with HAI activity against these H5-pseudotyped viruses to avoid potential interference that could influence measurements of NAI Ab titers.

### Hemagglutination inhibition (HAI) and microneutralization (MN) assays

Sera from ferrets infected with A/Cal/07/09 (obtained from BEI Resources; NR-15429) and patient sera collected at days 0 and 28 were tested in duplicate against A/Cal/04/09 and the virus isolated from patient 022 for hemagglutination-inhibiting (HAI) and HA-specific neutralizing Ab titers.

For HAI assays, twofold serial dilutions of human or ferret sera treated with receptor destroying enzyme (RDE) (Denka Seiken, Campbell, CA) were mixed with 8 HA units/50 μL of each virus. The HA protein of the A/Cal/04/09 virus used in the assays was identical to the one deposited in GenBank (accession number JF915184.1). The serum/virus mixture was incubated with 0.5% turkey red blood cells (Lampire Biological Laboratories, Pipersville, PA) for approximately 30 minutes at room temperature. The HAI Ab titer was measured as the highest dilution of serum at which hemagglutination was inhibited.

For MN assays, twofold serial dilutions of human or ferret sera was mixed with approximately 200 FFU of each virus and was left at room temperature for 60 minutes. Confluent MDCK cell monolayers were then inoculated with the serum/virus mixtures. After an adsorption period of 60 minutes at room temperature, serum/virus mixtures were removed and replaced with DMEM supplemented with 0.3% BSA, 10 μg/mL gentamycin, and 1 μg/mL TPCK-trypsin. After incubation for 8 hpi at 37°C in air enriched with 5% CO_2_, infected cells were fixed and permeabilized with 0.5% Triton X-100 and 4% formaldehyde in PBS, and an immunofocus assay was performed to determine the amount of not-neutralized virus. The HA-specific neutralizing Ab titer was measured as the highest dilution of serum at which more than 50% of FFU were inhibited.

### Recombinant influenza HA proteins

All recombinant (r)HA proteins, encoding a C-terminal trimerization domain and a hexahistidine purification tag [33] were cloned in the plasmid pFastBac CT-TOPO vector (Invitrogen, Grand Island, NY) and expressed using a baculovirus system, as previously described [34]. Purified rHAs were concentrated and desalted with 30 kDa Amicon Ultracell centrifugation units (Millipore, Billerica, MA) and re-suspended in PBS. The purity, integrity and identity of rHA proteins was assessed by NuPage 4–12% Bis-Tris gels (Invitrogen, Grand Island, NY) and Western blot. Western blot analysis was performed using polyclonal rabbit anti-influenza strain/subtype specific primary (eEnzyme, Gaithersburg, MD) and goat anti-rabbit horseradish peroxidase conjugated secondary (Bio-Rad, Hercules, CA) antibodies. Protein concentration was quantified using the Quickstart Bradford Dye Reagent (Bio-Rad, Hercules, CA) with a bovine serum albumin standard curve.

Purified rHA proteins for each influenza strain/subtype were covalently coupled to Bio-plex Pro™ Magnetic COOH Beads (Bio-Rad, Hercules, CA) using the Bio-Plex Amine Coupling Kit (Bio-Rad, Hercules, CA) as previously described [34]. The coupled beads were tested with anti-HA subtype specific ferret or rabbit polyclonal antibodies (Influenza Reagent Resource, IRR, Manassas, VA) and a goat anti-rabbit or anti-ferret phycoerythrin (PE)-conjugated detection Ab (SouthernBiotech, Birmingham, AL).

### mPlex-Flu assay

Quantitation of Ab levels in human sera was performed as previously described [34]. Briefly, a panel of HA-coupled mPlex-Flu beads were mixed and incubated with diluted human serum, at 500 beads per each HA to be detected, in 96-well filtration plates (Millipore, Billerica, MA) at 4°C overnight, on a rotary shaker (500 rpm), in the dark. Wells were washed twice and then incubated with PE-conjugated anti-mouse IgG (γ chain specific, at 1:400 dilution) secondary antibodies (SouthernBiotech, AL) in the dark at room temperature for 2 h with gentle agitation (500 rpm). After 3 additional washes, the beads in each well were resuspended in Luminex Magpix Drive Fluid (Luminex, Austin, TX), analyzed on a Magpix multiplex reader (Luminex, Austin, TX). The results were expressed as median fluorescence intensity (MFI). The infected subject’s sera were diluted 1: 5,000 and 1:10,000 and the absolute concentration of anti-HA antibodies present in serum were calculated from a standard curve which was generated at the same time as previously described [34]. Average concentrations of each anti-HA antibody from both serum dilutions was shown in the heatmaps.

### Enzyme-linked lectin assay (ELLA)

Sera from ferrets infected with A/Cal/07/09 (BEI Resources; NR-15429) and patient sera collected at days 0 and 28 were tested in duplicate against sciIAV-NA-wt and sciIAV-NA-001 for NAI Ab titers using a modified enzyme-linked lectin-based assay (ELLA), as previously described [19,35,36]. Bound lectin was detected with 3,3',5,5'-tetramethylbenzidine (TMB) (Thermo Scientific #34028), and optical density was measured at 450 nm. Each virus was titrated by ELLA using 10-fold serial dilutions in the absence of serum, and the dilution for each virus at which the optical density at 450 nm was closest to the maximum signal but within the linear range of the titration curve was selected to maximize assay sensitivity and precision.

At least 8 wells on each plate were dedicated to positive (no serum; virus only) or negative (blank; no serum or virus) controls. The average background signal (negative control) was subtracted from all readings, and percent inhibition compared to the average maximum signal (positive control) at each serum dilution was calculated. The NAI Ab titer was measured as the highest dilution at which at least 50% of the maximum signal was inhibited. Only assays in which the background values were less than 10% of the maximum signal were considered.

### Publically available HA and NA sequences

19,926 HA and 17,430 NA DNA sequences of pH1N1-like strains isolated from humans since 2009 were downloaded from the GISAID online database at the time of investigation (http://platform.gisaid.org/epi3/frontend). 16,981 HA and 15,796 NA DNA sequences remained after removing sequences lacking associated metadata, or containing missing or erroneous (not A, C, T, or G) nucleotides. The HA (9) and NA (9) sequences from the Rochester, NY viral isolates were added to the publically available sequences, resulting in 16,990 HA and 15,805 NA DNA sequences which were used for all sequence-based analyses. Sequences were aligned using MUSCLE [37,38].

### Phylogenetic reconstructions

Phylogenies were reconstructed with the neighbor-joining method and bootstrapped 1000 times with ClustalX version 2.1, using 16,990 HA or 15,805 NA aligned DNA sequences [39]. Each individual branch of the phylogenetic trees were colored according to the season during which the strain encoding each HA or NA protein was isolated. Phylogenetic trees were rendered in FigTree (http://tree.bio.ed.ac.uk/software/figtree/). Clades were identified as previously described [8].

### Selection analyses

A single-likelihood ancestor counting (SLAC) approach [40] was used to infer the non-synonymous (dN) and synonymous (dS) substitution rates on a per-site basis under the HKY85 evolutionary model for 16,990 HA or 15,805 NA aligned DNA sequences encoded by pH1N1-like strains and phylogenies reconstructed using ClustalX (see above) with HyPhy MPI [41]

### Sequence-based mapping

For each HA sequence, we calculated its hamming distance to all other HA sequences in the data set, as previously described [42]. The hamming distance is the number of amino acid differences at each residue’s position in the hemagglutinin protein. This process results in a distance matrix consisting of the number of amino acid differences between all viruses in the data set. Classical multidimensional scaling (principal coordinates analysis) was performed on the distance matrix using the *stats* package in R.

### Statistical analyses

Log2 transformation was applied to Ab titers to stabilize data variability and normalization. Repeated ANOVA was used to simultaneously study multiple effects (string and time), and to take account of within-subject correlations. Multiple testing adjustment of Tukey method was applied to prevent type I error inflation. The findings were confirmed by pairwise comparisons through Sign test.

## Results

### The HA and NA proteins encoded by 2015–2016 influenza virus isolates from Rochester, NY encode amino acid differences compared to the strain used for vaccination that same season

Influenza virus HA and NA proteins undergo antigenic drift, leading to decreased vaccine efficacy [21,43,44]. Previously, it was shown that risk of infection with influenza during the 2010–2011 and 2012–2013 flu seasons may have been due to a poor response to vaccination as well as contact with antigenically distinct IAV strains [26]. To analyze genetic and antigenic variation within the HA and NA proteins of pH1N1-like viruses circulating during the 2015–2016 season, 14 nasal swabs/washes were collected in Rochester, NY. All mutations described are numbered by their position along the amino acid sequence relative to the methionine codon at the beginning of the N-terminal signal peptide (position 1). All comparisons, both sequence-based and serological assays, were performed using A/Cal/04/09, another early isolate, as the reference strain [45]. H1N1 viruses emerging in 2009 were antigenically homogenous [46]. The HA protein sequence of A/Cal/04/09 differs from that of A/Cal/07/09 by 2 non-synonymous mutations at positions 200 and 214, and the NA protein sequences of the two strains are identical (see Tables 1 and 2, respectively). With these differences taken into consideration, A/Cal/04/09 was used to represent the vaccine strain throughout this paper.

**Table 1:** Mutations present in the HA protein of 2015–2016 circulating pH1N1-like strains compared to the vaccine strain.

			Head domain												Stalk domain				
					Sa		Sb				Ca1													
Amino Acid residue*	13	100	101	114	179	180	200	202	207^#^	214	220	233	273	300	338	391	468	472	516
A/California/04/2009	A	P	S	D	S	K	P	S	S	T	S	I	A	K	I	E	S	N	E
A/California/07/2009	A	P	S	D	S	K	S	S	S	A	S	I	A	K	I	E	S	N	E
ACU 001	T	S	N	N	N	Q	S	T	S	A	T	T	T	E	V	K	N	N	K
ACU 004	T	S	N	N	N	Q	S	T	S	A	T	T	T	E	V	K	N	T	K
ACU 005	T	S	N	N	N	Q	S	T	S	A	T	T	T	E	V	K	N	N	K
ACU 007	T	S	N	N	N	Q	S	T	R	A	T	T	T	E	V	K	N	N	K
ACU 008	T	S	N	N	N	Q	S	T	S	A	T	T	T	E	V	K	N	N	K
ACU 009	T	S	N	N	S	Q	S	T	S	A	T	T	T	E	V	K	N	N	K
ACU 012	T	S	N	N	N	Q	S	T	S	A	T	T	T	E	V	K	N	N	K
ACU 017	T	S	N	N	N	Q	S	T	R	A	T	T	T	E	V	K	N	T	K
ACU 022	T	S	N	N	N	Q	S	T	S	A	T	T	T	E	V	K	N	N	K

*Amino acid substitutions found in the HA protein of circulating pH1N1-like viruses compared to the HA proteins of A/Cal/04/09 and A/Cal/07/09. All mutations are numbered by their position along the amino acid sequence relative to the methionine codon at the beginning of the N-terminal signal peptide (position 1). Location of mutations with respect to head domain, stalk domain, and known Ab-binding sites is indicated. Gray colors indicate amino acid residues 179, and 180 in the Sa antigenic site; 200, and 202 in the Sb antigenic site; and 220 in the Ca1 antigenic site. The “acronym” ACU derives from acute (ACU) infection.

^#^Amino acid changes (position 207) are introduced only after growing viruses isolated from patients 007, and 017 in MDCK cells. These mutations were not detected in the viruses present in the nasal washes/swabs of these patients.

**Table 2:** Mutations present in the NA protein of 2015–2016 circulating pH1N1-like strains compared to the vaccine strain.

Amino Acid residue*	13	34	40	44	200	241	248	264	270	314	321	369	386	432
**A/California/04/2009**	**V**	**I**	**L**	**N**	**N**	**V**	**N**	**V**	**N**	**I**	**I**	**N**	**N**	**K**
A/California/07/2009	V	I	L	N	N	V	N	V	N	I	I	N	N	K
ACU 001	I	V	I	S	S	I	D	I	K	M	V	K	K	E
ACU 004	I	V	I	S	S	I	D	I	K	M	V	K	K	E
ACU 005	I	V	I	S	S	I	D	I	K	M	V	K	K	E
ACU 007	I	V	I	S	S	I	D	I	K	M	V	K	K	E
ACU 008	I	I	I	S	S	I	D	I	K	M	V	K	K	E
ACU 009	I	V	I	S	S	I	D	I	K	M	V	K	K	E
ACU 012	I	V	I	S	S	I	D	I	K	M	V	K	K	E
ACU 017	I	V	I	S	S	I	D	I	K	M	V	K	K	E
ACU 022	I	I	I	S	S	I	D	I	K	L	V	K	K	E

* Mutations found in the NA protein of circulating pH1N1-like viruses compared to the NA protein of A/Cal/04/09 and A/Cal/07/09. All mutations are numbered by their position along the amino acid sequence relative to the methionine codon at the beginning of the N-terminal signal peptide (position 1). Location of mutations with respect to known Ab-binding sites is indicated. Gray indicates mutations in known Ab-binding sites. The “acronym” ACU derives from acute (ACU) infection.

Influenza viruses present in nasal washes/swabs from subjects infected during the 2015/2016 season encoded 18 amino acid changes in the HA protein compared to the strain Cal/04/2009, circulating at the origin of the 2009 pandemic (Table 1). The infecting viruses were isolated in MDCK cells from 9 out of the 14 nasal swabs/washes and HA sequences were obtained from the viruses (Table 1). The HA sequences of viruses grown in MDCK cells were the same as those of viruses present in the original nasal washes/swabs (data not shown), with the exception of viruses isolated from patients ACU007 and ACU017, that introduced an additional mutation S207R (Table 1). As with the majority of H1N1 viruses circulating during the 2015–2016 season, these isolates were classified as clade 6B.1 [8,25].

**Fig 1:**
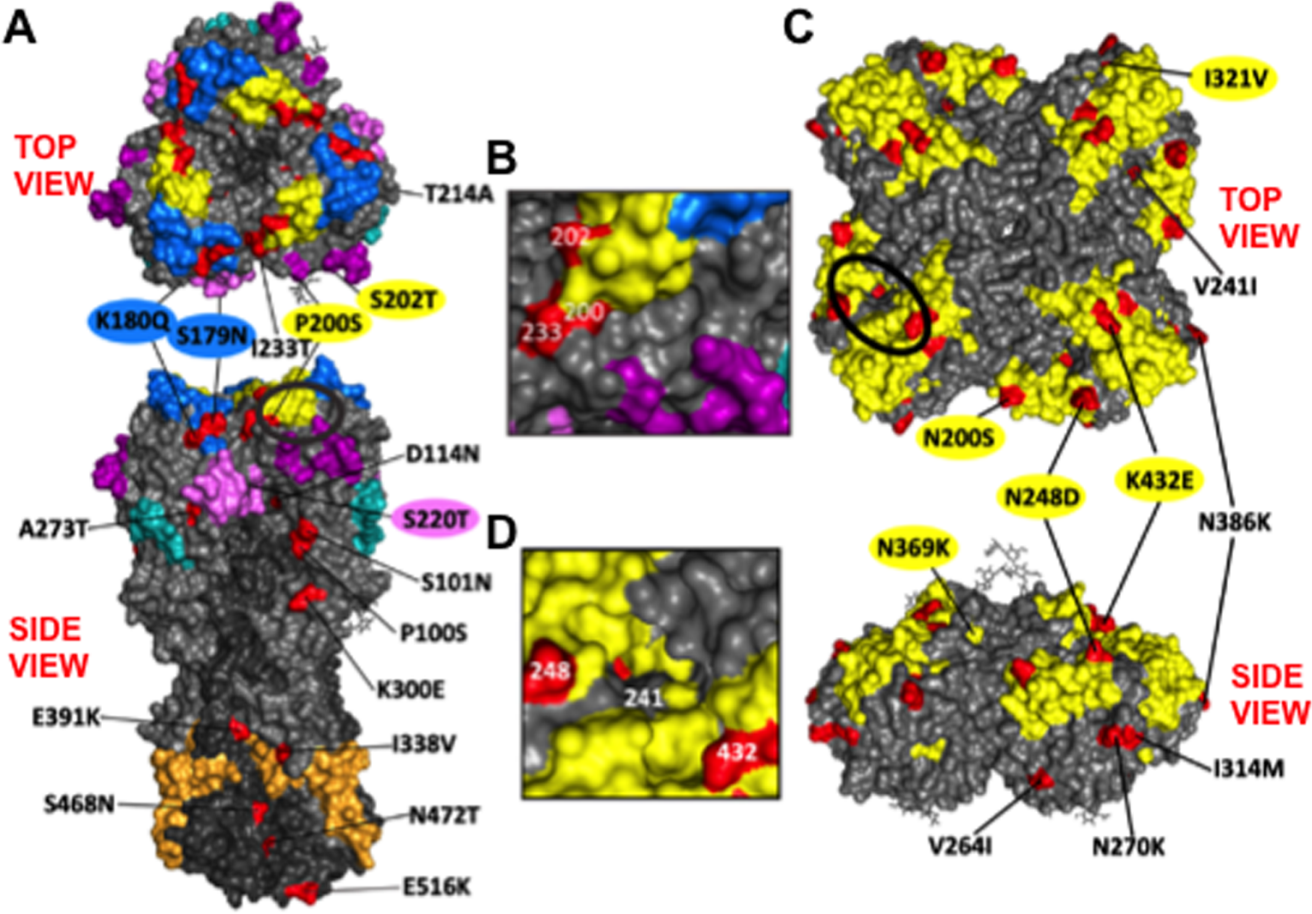
Mutations present in the HA or NA proteins of 2015–2016 circulating pH1N1-like strains compared to the vaccine strain. Three-dimensional models of the A/Cal/04/09 HA (A and B) or NA (C and D) proteins (PDB ID: 3LZG and 3NSS, respectively), showing the mutations of circulating pH1N1-like viruses compared to that of the vaccine strain. A top and views of the HA or NA protein quaternary structures are shown. The HA antigenic sites Sa (dark blue), Sb (yellow), Ca1 (light purple), Ca2 (dark purple), and Cb (light blue) are indicated. The HA fusion domain is represented in orange [52,53]. A bracket (not to scale) indicates the location of the NA stalk had it been resolved in the crystal structure. Known antigenic sites on the NA protein are represented in yellow. The mutations present in the HA or NA of pH1N1-like circulating strains compared to that of the H1N1 vaccine strain are shown in red. The approximate location of HA mutation S220T, situated in the Ca1 antigenic site at the subunit interface, is indicated. The ellipse indicates the receptor-binding site (RBS) of the HA (B) or the catalytic site of the NA (D), shown at a higher magnification. 3D model was customized using PyMOL.

Out of these 18 mutations, 7 were located in the HA stalk domain while the other 11 were located in the HA head domain (Table 1). As shown on a 3D model of the HA protein (RCSB PDB ID: 3LZG) [47], 5 mutations were found in previously characterized [4] Ab-binding sites: S179N and K180Q, in Sa; P200S and S202T, in Sb; and S220T, in Ca1 (Fig 1A and Table 1) [9]. Three of the mutations (P200S and S202T close to the 190 alpha helix, and I233T along the 220 loop) were in close proximity to the receptor-binding domain (Fig 1B) [3,5,48]. The 190 helix and the 220 loop are numbered with respect to the structure-based sequence alignment of different HA subtypes based on H3 numbering [49]. All isolated viruses encoded the same HA head domain except for virus ACU009, which lacked the S179N mutation. Additionally, there were variations in the HA stalk domain between the viruses infecting the 9 patients, as only two viruses (infecting patients ACU004 and 017) encoded substitution T472N (Table 1).

As for the NA protein (sequenced from nasal swabs/washes, as well as the 9 infecting viruses isolated in MDCK cells), 14 mutations were found when compared to the vaccine strain (Table 2). As seen on a 3D model of the NA protein (RCSB PDB ID: 3NSS) [50], 5 out of the 14 mutations (N200S, N248D, I321V, N369K, and K432E) were at previously characterized Ab-binding sites (Fig 1C) [51]. Three of the mutations (K432E along the 430 loop, V241I and N248D) were located near the catalytic site (Fig 1D). The 430 loop is numbered with respect to structure-based sequence alignments based on N2 numbering [50]. All isolates encoded the same NA sequence, with the exception of viruses infecting patients ACU008 and 022, which did not encode the mutation I34V. Altogether, these data indicate genetic variability in both, the HA and NA proteins of currently circulating H1N1 viruses, compared to the vaccine strain.

### The HA and NA proteins encoded by influenza virus isolated in Rochester (NY) are representative of the HA and NA proteins from viruses circulating worldwide during the 2015–2016 season

To analyze if the HA and NA protein sequences of viruses isolated in the Rochester, NY area, were representative of viruses circulating globally, these HA and NA sequences were compared to those of pH1N1-like viruses isolated worldwide during the 2015–2016 flu season. Out of the 6,552 HA and 5,462 NA sequences of 2015–2016 isolates uploaded to the GISAID database at the time of investigation, the majority of isolates from different geographic regions encoded HA and NA proteins with mutations identical to those described here (S2 and S3 Tables, respectively). These data indicate that the HA and NA proteins encoded by viruses isolated in Rochester, NY (Tables 1 and 2, respectively), are representative of those encoded by the dominant pH1N1-like strains circulating during the 2015–2016 season in all geographic regions (S2 and S3 Tables, respectively).

### pH1N1-like HA and NA glycoproteins show directional selection of mutations in antigenic sties

**Fig 2:**
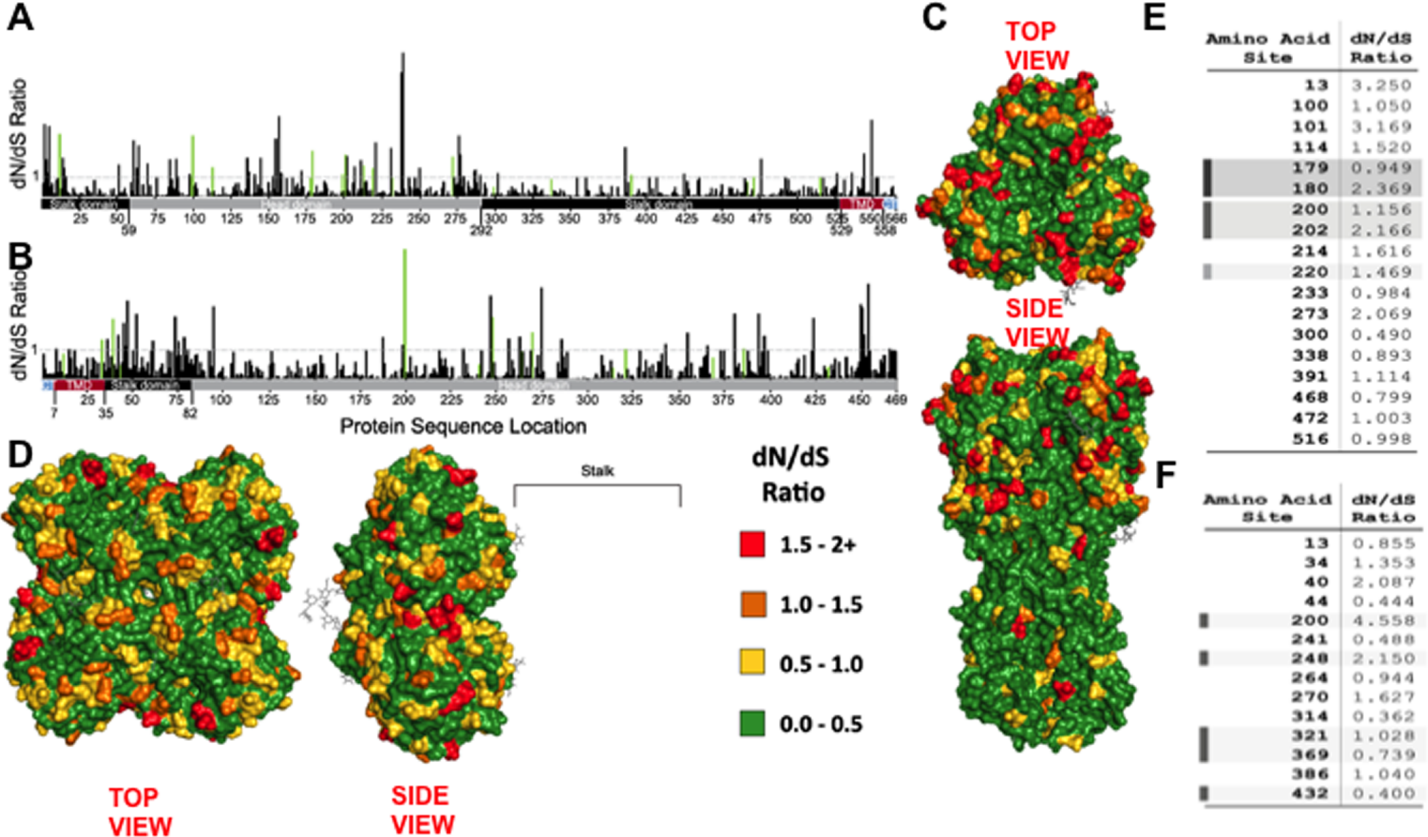
dN/dS selection analysis for pH1N1-like HA or NA proteins. A selection analysis (dN/dS ratio) was performed on 16,990 HA (A and C) or 15,805 NA (B and D) DNA sequences of pH1N1-like strains isolated from humans since 2009, and the dN/dS ratio at each codon along the HA (A) or NA (B) DNA sequence was plotted. The dN/dS ratio for codons at which mutations were found in 2015–2016 isolates are shown in green. The lower bar represents amino acid residues along the HA or NA proteins corresponding to the head domain (gray), the stalk domain (black), the transmembrane domain (TMD) (red), and the cytoplasmic tail (CT) (blue). Three-dimensional model of the HA (PDB ID: 3LZG) (C) or the NA (PDB ID: 3NSS) (D) proteins, with amino acid residues colored according to the dN/dS ratio at the corresponding codon using PyMOL. A bracket (not to scale) indicates the location of the NA stalk had it been resolved in the crystal structure. (E and F) Tables showing the dN/dS ratio for the codons at which amino acid substitutions were present in the HA (E) or NA (F) of pH1N1-like viruses circulating during 2015–2016. Gray shadows indicate codons at positions within known Ab-binding sites.

To analyze selection pressures at the sites of mutations present in circulating strains, selection analyses (dN/dS ratio) [54–56] were performed on 16,990 HA or 15,805 NA DNA sequences of pH1N1-like strains isolated from humans since 2009. The dN/dS ratio is the ratio of the rate of non-synonymous mutations (dN) to the rate of synonymous mutations (dS) at each codon. As such, dN/dS has been used as a measure of evolutionary change by giving an estimate of the magnitude and direction (positive or negative) of selective pressure acting on a codon [41,55,57,58]. Of the 566 amino acids comprising the HA protein of pH1N1-like strains, 12.7% (72 of 566) of the codons were subject to positive (diversifying) selection (dN/dS > 1), while 87.3% of codons (494 of 566) were under negative (purifying) selective pressure (dN/dS < 1) (Fig 2A and 2C) [41,55]. There were more codons under positive selective pressure in the HA head region (19.7%) than the HA stalk region (7.8%). Interestingly, 12 of the 18 amino acids (positions 13, 100, 101, 114, 180, 200, 202, 214, 220, 273, 391, and 472), at which mutations were found in the HA of 2015–2016 isolates, were under positive selective pressure (Fig 2E), suggesting that these mutations are the result of positive (diversifying) selection. Four of these amino acids are located in antigenic sites in the HA.

Of the 469 amino acids in the NA protein of pH1N1-like strains, 11.3% (53 of 469) of codons were subject to positive selection, while 88.7% of codons (416 of 469) were under negative selective pressure (Fig 2B and 2D). Interestingly, seven of the 14 amino acid residues (positions 34, 40, 200, 248, 270, 321, and 386) at which mutations were found in the NA of 2015–16 isolates, three of which are located in antigenic sites, were under positive selective pressure (Fig 2F).

**Fig 3:**
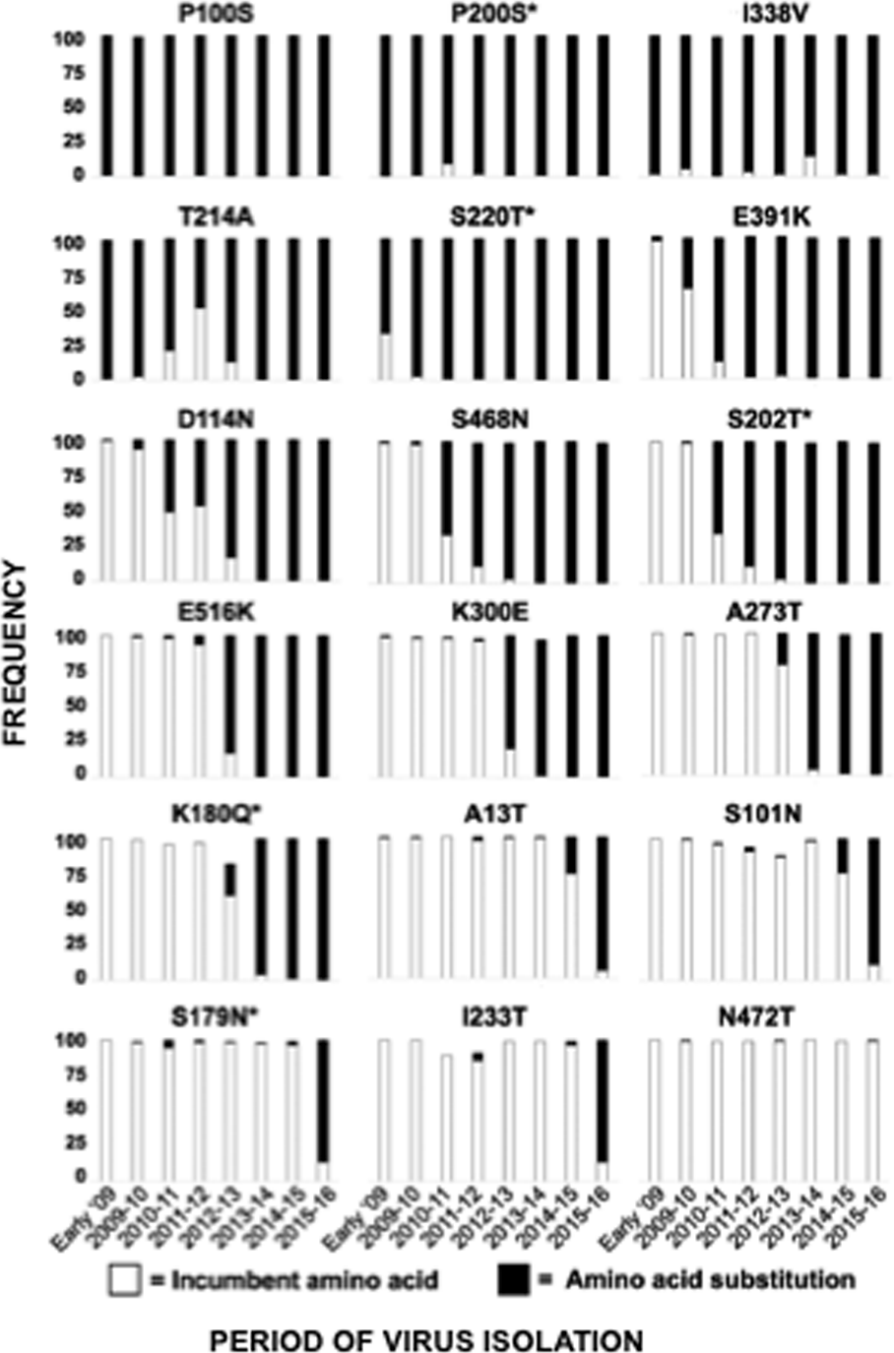
Frequency of HA amino acid changes found in pH1N1-like viruses over time. Publicly available sequences of the HA protein of pH1N1-like strains isolated since 2009 (n = 16,990) were analyzed, and were plotted according to the percentage of sequences containing the original (incumbent) amino acid present in the H1N1 vaccine strain (white) or the substitute amino acid (black) in pH1N1-like isolates at positions 100, 200, 338, 214, 220, 391, 114, 468, 202, 516, 300, 273, 180, 13, 101, 179, 233, 472 from each season since early 2009. An (*) next to the amino acid location indicates that the position is in a previously described antigenic site.

**Fig 4:**
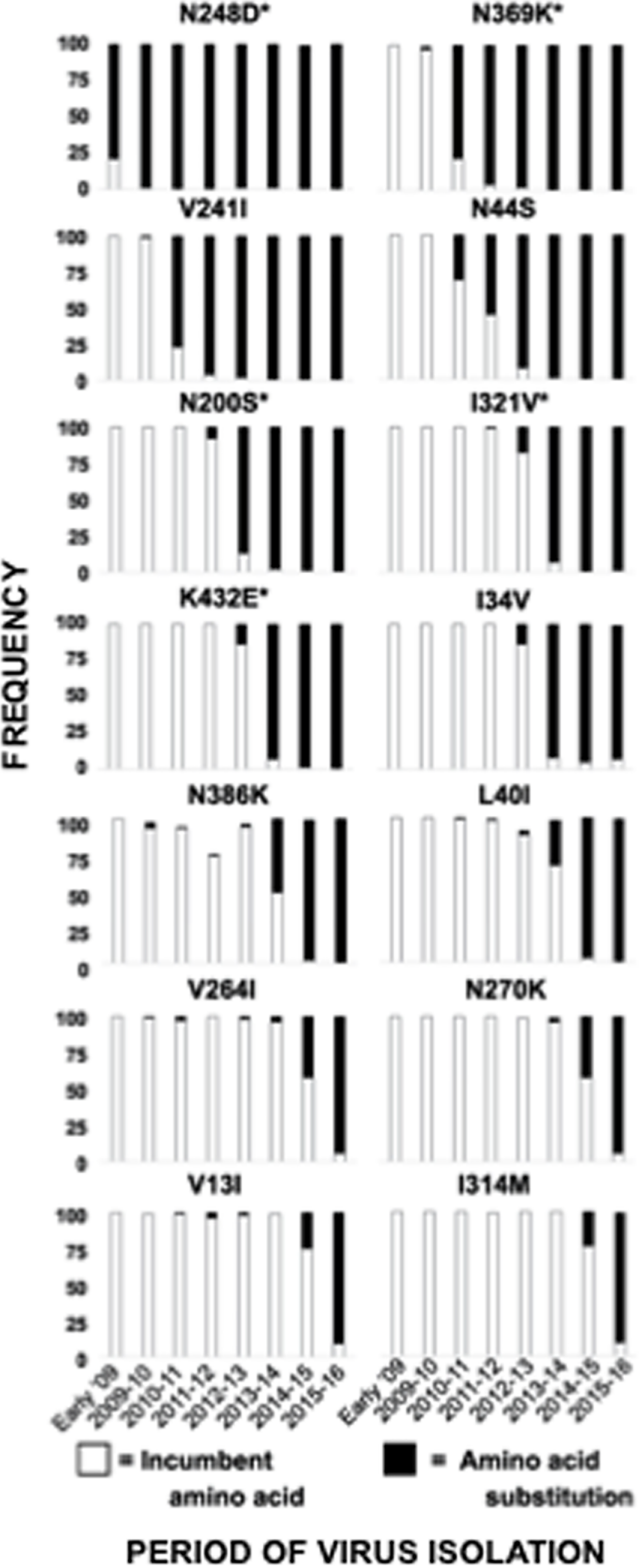
Frequency of NA amino acid changes found in pH1N1-like viruses over time. Publicly available sequences of the NA protein of pH1N1-like strains isolated since 2009 (n = 15,805) were analyzed, and were plotted according to the percentage of sequences containing the original (incumbent) amino acid present in the H1N1 vaccine strain (white) or the substitute amino acid (black) in pH1N1-like isolates at positions 13, 34, 40, 44, 200, 241, 248, 264, 270, 314, 321, 369, 386, and 432 from each season since early 2009. An (*) next to the amino acid location indicates that the position is in a previously described antigenic site.

**Fig 5:**
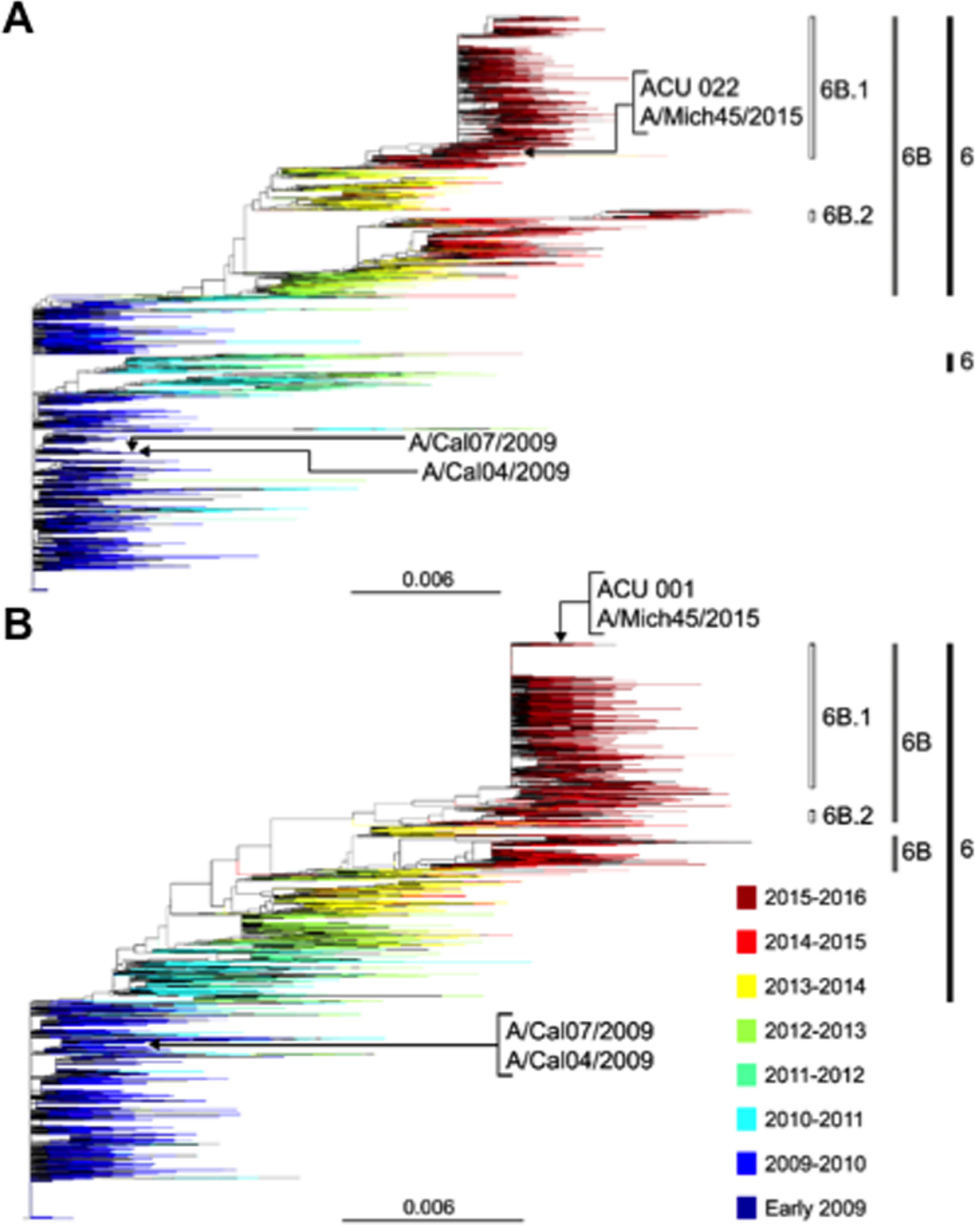
The genetic relatedness of the HA and NA proteins encoded by pH1N1-like viruses circulating since 2009. Phylogenetic reconstruction of HA (A, n = 16,990) and NA (B, n = 15,805) proteins encoded by pH1N1-like viruses. Clades are indicated to the left; branches are colored according to season of isolation.

As expected, mutations have been incorporated by the pH1N1-like virus population over the past seven seasons, beginning with the emergence of the pandemic-causing triple reassortant in early 2009 [8,59,60]. Notably, strains encoding an HA protein with five of the mutations (P100S, P200S, S220T, I338V, and T214A) became the dominant circulating strains early in 2009 following detection of the outbreak (Fig 3). With respect to the NA protein, only one mutation, N248D, appeared in the NA protein of circulating strains immediately following the outbreak in early 2009 (Fig 4). The remaining mutations in the HA and NA proteins were introduced in strains circulating during following seasons (Figs 3 and 4). Importantly, once each mutation was introduced into the HA (Fig 3) or NA (Fig 4) by pH1N1-like strains, the percentage of total pH1N1-like isolates encoding the mutation increased each season and remained prevalent in isolates of following seasons. The order in which mutations were introduced corresponds with the phylogenetic analysis of the HA (Fig 5A) or the NA (Fig 5B) of pH1N1-like strains performed by us as well as others [8,59,60]. These data suggest evolution and selection pressure on the HA and NA proteins from pH1N1 viruses circulating since 2009.

### The mutations in the HA protein encoded by circulating viruses contribute to the evasion of HA-binding antibodies

**Fig 6:**
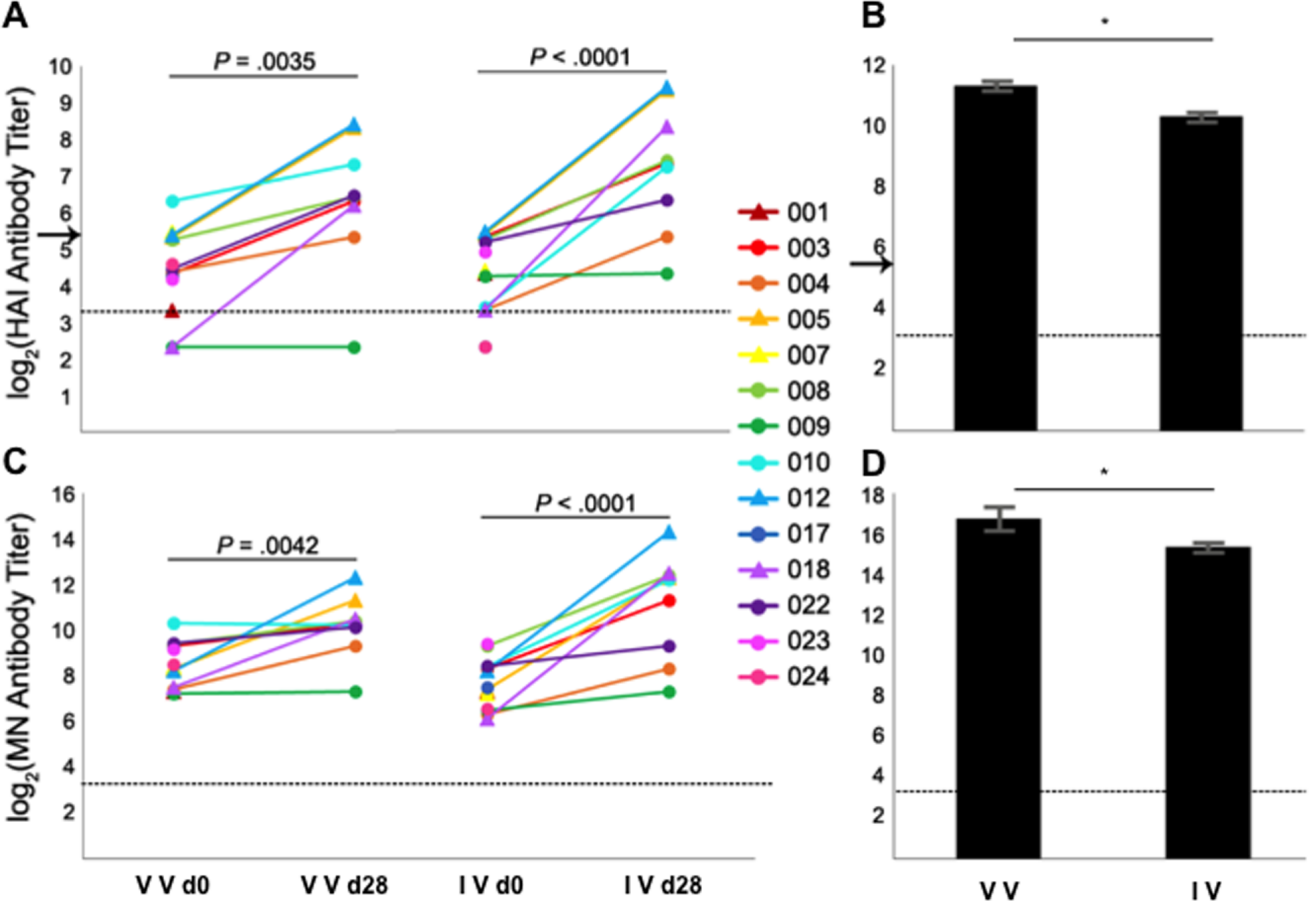
Effect of mutations present in the HA ofin 2015–2016 isolates on the antigenicity of the viral protein. HAI (A and B) and MN (C and D) assays using human (A and C) and ferret (B and D) antisera. Standardized antisera from ferrets infected with the vaccine virus, and human sera collected from infected subjects at the acute (day 0) and post-acute visits 28 days later (day 28), were measured using HAI or MN assays for antibodies specific for the H1N1 vaccine strain (VV) and the virus isolated from patient 022 (IV). The HA protein of the virus isolated from patient 022 encodes mutations found in the majority of 2015–2016 isolates circulating worldwide (S2 Table and Table 1). Individual subject numbers from which the viruses were isolated are shown in the legend. Subjects whose titers are represented with circles received the 2015–2016 flu vaccine, while those represented with triangles were not vaccinated (see S1 Table). Experiments were repeated three times, showing reproducible data. *, indicates p-values <0.05 using a Student’s t-test in B and D. IV, isolated virus. VV, vaccine virus. HAI Ab titer of 40, commonly associated with protection, is indicated by an arrow in (A) and (B) [62,63]. The dotted line indicates the limit of detection (LoD), at an Ab titer of 10. A titer of 5 was considered for titers that fell below the (LoD).

HA is the main viral protein inducing antibodies that protect against infection [61]. To analyze the relevance of the mutations in the HA protein in evading serum antibodies induced by previous exposure/vaccination, HAI and MN assays were performed using serum from ferrets raised against A/Cal/07/09 and human sera obtained from 14 patients during the acute visit (approximately 2–3 days after infection; day 0) and the post-acute visit (28 days after the acute visit; day 28, not available for patients 001, 007, 017, 023, and 024) (Fig 6). Virus isolated from patient 022 was used and referred to as the circulating strain, as it comprises the mutations common to the HA of pH1N1-like strains isolated worldwide during the 2015–2016 flu season (S2 Table and Table 1). Given that sera was obtained from patients who were infected, it was not surprising that HAI Ab titers were low (< 40, in 9 out of 14 patients) in human sera collected at day 0, even in patients who were vaccinated that season (patients 003, 004, 008, 009, 010, 017, 022, 023, and 024), and only one subject (010) showed a titer >40 (Fig 6A). These data showing low HAI titers even in vaccinated subjects suggests poor responses to the pH1N1 component of the vaccine. Globally, at day 28, HAI Ab titers significantly (*P*<0.005) increased in patient serum, as expected [26], with the exception of patient 009. At day 0, HAI Ab titers using human sera from patients 005, 008, and 012 were similar for both circulating and vaccine strains (Fig 6A). However, HAI Ab titers in the sera of patients 004, 007, 010, 017, and 024 at day 0, and in serum from ferrets infected with A/Cal/07/09 virus (Fig 6A and 6B) were demonstrably lower (at least 2-fold) for the circulating strain than for the vaccine strain, showing that the 18 mutations found in the viruses which circulated during the 2015–2016 season affected HA protein antigenicity to a limited degree.

In MN assays, serum Ab titers specific for the infecting and vaccine strains were significantly (*P*<0.005) higher after infection (day 28), as expected. The only exception was the titers specific for the vaccine strain, which did not increase in patients 009 and 010 (Fig 6C). Interestingly, at day 0, HA-specific neutralizing Ab titers in serum from patients 003, 004, 005, 007, 009, 018, and 022 were 2-fold lower for the circulating strain than for the vaccine strain, and neutralizing Ab titers in the serum of patients 010, 017, and 024 were 4-fold lower for the circulating strain than for the vaccine strain (Fig 6C). Using ferret serum, we observed over 2-fold difference in neutralizing Ab titers specific to the vaccine strain compared to the circulating strain (Fig 6D). Taken together, the data from the HAI assays and the MN assays indicate that at least one of the mutations found in the HA of circulating viruses make a measurable but limited contribution to the evasion of HA-Ab binding.

### The Ab response after infection is adapted to the HA of the pH1N1-like infecting virus

**Fig 7:**
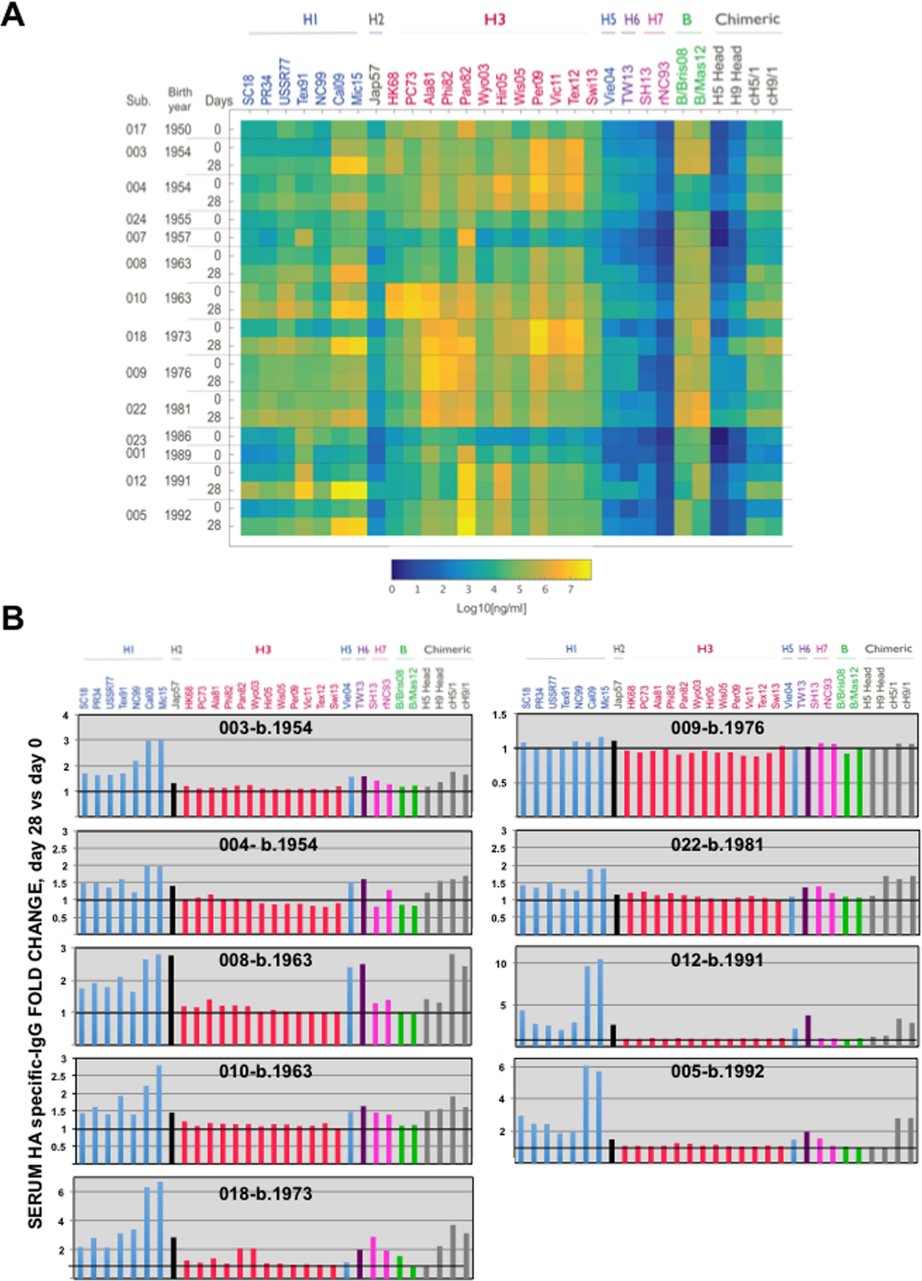
Binding of serum from pH1N1-infected subjects to HA proteins from different strains. (A) Concentrations of sera IgG antibodies cross-reacting to each HA protein were measured by multiplex assay against the indicated IAV and IBV HA proteins. Heatmap showing the average Ab concentrations using two human sera dilutions (1:5,000 and 1:10,000) in duplicates. Subject number, subject year of birth and visit of sera extraction (d0 or d28) are indicated on the left. (B) Graphs representing the fold-change in binding to HA proteins using the sera from day 0, compared to the sera from day 28. Each graph represents one subject. For the HA protein strains: Blue colors represent H1N1 strains (A/South Carolina/1/1918, SC18; A/Puerto Rico/8/1934, PR8; A/USSR/90/1977, USSR77; A/Texas/36/1991, Tex91; A/New Caledonia/20/1999, NewCal99; A/California/04/2009, Cal09; A/Michigan/45/2015, Mic15), black color represents an H2N2 strain (A/Japan/305/1957, Jap57), pink colors represent H3N2 strains (A/HongKong/1/1968, HK68; A/Port Chalmers/1/1973, PC73; A/Alabama/1/1981, Ala81; A/Philippines/2/1982, Phi82; A/Panama/7/1999, Pan99; A/Wyoming/2003, Wyo03; A/Hiroshima/52/2005, Hir05; A/Wisconsin/67/2005, Wis05; A/Perth/16/2009, Perth09; A/Victoria/361/2011, Vic11, A/Texas50/2012, Tex12, A/Switzerland/2013, Swit/2013), blue color represents H5N1 (A/Vietnam/1204/2004, Viet04), purple color represents H6N1 (A/Taiwan/2/2013, TW13), fucsia colors represent H7N9 (A/Shanghai/2/2013, SH13 and H7N1 (A/rhea/North Carolina/39842/93, rhea/NC93), green color represent B (B/Brisbane/60//2008, Bris08; B/Phuket/2013, Phu13), and gray colors represent H5, and H9 head, and chimeric proteins cH5/1 and cH9/1.

Circulating levels of HA-reactive Ab levels in humans are often highest against HA proteins related to those of viruses that caused significant early-life influenza virus exposure. Generally, later infections/vaccinations maintain these patterns long-term by preferential boosting of cross-reactive anti-HA Ab levels, a phenomenon known as “original antigenic sin (OAS)” (reviewed in [64]). To relate the Ab response to the HA of the infecting virus to OAS-type production of H1-reactive Abs, we used a multiplex assay to measure the levels of Abs reactive to a range of H1 variants (Fig 7). Binding was also measured against a range of H3 variants, other influenza A HA subtypes, influenza B HAs, and chimeric proteins (cH5/1 and cH9/1). The chimeric proteins, encoding “exotic” H5 or H9 head domains and a A/Cal/04/09 HA stalk domain were included to preferentially measure the induction of antibodies specific for the HA stalk domain after infection. A sequence-based analysis was performed to visualize the amino acid variability among the different HA subtypes, and among the different H1 strains, as previously described [42] (S1A and S1B Fig). Importantly, the HA protein from A/Michigan/45/2015 H1N1 strain encoded the same amino acid sequence as the virus isolated from patient 022, used for the HAI and MN assays. All the sera from pH1N1-like virus-infected subjects extracted at day 0 (acute visit) showed low binding to A/Cal/04/09 and A/Michigan/45/2015 HA proteins (Fig 7A), being the sera from patient 010 the one showing the highest (but still low) binding (Fig 7A). These results are consistent with the low HAI titers detected in HAI assays, in which the highest titer at day 0 (80) was observed for this same patient 010 (Fig 6A). Antibodies specific to the HA proteins from A/Cal/04/09 and A/Michigan/45/2015 increased from 1.9 to 10-fold in all the subjects at day 28, compared to day 0, with the exception of patient 009 (Fig 7A and 7B), as observed in the HAI assays (Fig 6A). In addition, whereas at day 0 subject 010 sera bound A/Cal/04/09 HA protein to a higher degree than A/Michigan/45/2015 (as shown in Fig 6A for the HAI titers), at day 28, human sera from subjects 004, 005, 012, 018, and 022 showed a slightly higher reactivity to the HA protein from A/Michigan/45/2010 strain than to the HA protein from A/Cal/04/09 strain (Fig 7A). These data are consistent with an immune response adaptation to the infecting virus, and indicated that the HA proteins from A/Cal/04/09 and A/Michigan/45/2015 (encoding the same amino acid sequence to the HA protein from most 2015/2016 circulating strains) are not antigenically identical since different human sera do not bind both of them to the same extent.

Serum Ab binding to the chimeric HAs cH5/1 and cH9/1 increased more than 1.5-fold in 8 of 9 subjects, indicating induction of Abs reactive to the stalk region of H1 (a group 1 HA) (Fig 7A and 7B). In most of the subjects, there was an slight increase in Ab binding to other group 1 HAs (H2, H5, and H6), consistent with production of Abs specific for the conserved stalk (Fig 7A and 7B), as it has been shown in other studies demonstrating an anti-HA stalk Ab response following influenza infection in humans [17,65,66]. Ab binding to the group 2 HAs H3 and H7 changed very little or nothing from days 0 to 28 (Fig 7A and 7B), indicating that pH1N1 infection generates little anti-stalk Ab that cross-reacts between HA groups, and likely reflecting that the HA stalk domain differs significantly between the two groups of HA proteins [67].

Three subjects had relatively high day 0 Ab levels against H1s of older H1N1 viruses that were consistent with OAS-type patterns: subjects 010 (b. 1963) and 018 (b. 1973) had high day 0 titers against H1 USSR77, and subject 012 (b. 1991) had high titers against H1 Tex91 (Fig 7A). In most subjects, H1N1 infection (day 28 sera) increased day 0 Ab levels against older H1s and maintained the day 0 binding profiles, consistent with production of Abs that cross-reacted with H1 variants. The magnitude of these increases was not accounted for by increased levels of stalk-specific Abs (Fig 7B), indicating that the cross-reactive response was directed at HA head epitopes. Notably, the most marked increase in HA-binding Ab using sera collected at day 28 was against the H1s of A/Cal/04/09 and A/Michigan/45/2015 (Fig 7B), indicating that OAS-type responses were not at the expense of Ab adaptation to the infecting virus. OAS-type patterns of H3-binding Abs on day 0 were evident in most subjects, but these levels remained largely unchanged after H1N1 infection. For example, using sera from day 0, we found that patient 010 (b. 1963) preferentially recognized proteins from H3N2 viruses circulating in 1968 (HK68), 1973 (PC73) and 1982 (Phi82). Patients 018, 009 and 022 (b. 1973, 1976, and 1981 respectively) preferentially recognized the H3 proteins from 1981 and 1982 strains. Patients 001 and 005 (b.1989 and 1992, respectively) recognized best the H3 protein from A/Panama/99 than the H3 proteins circulating before 1992 and after 1999, likely reflecting a first exposure to a similar A/Pan99 strain (Fig 7A).

### Mutations in the NA protein encoded by circulating viruses contribute to the evasion of NA-binding antibodies

**Fig 8:**
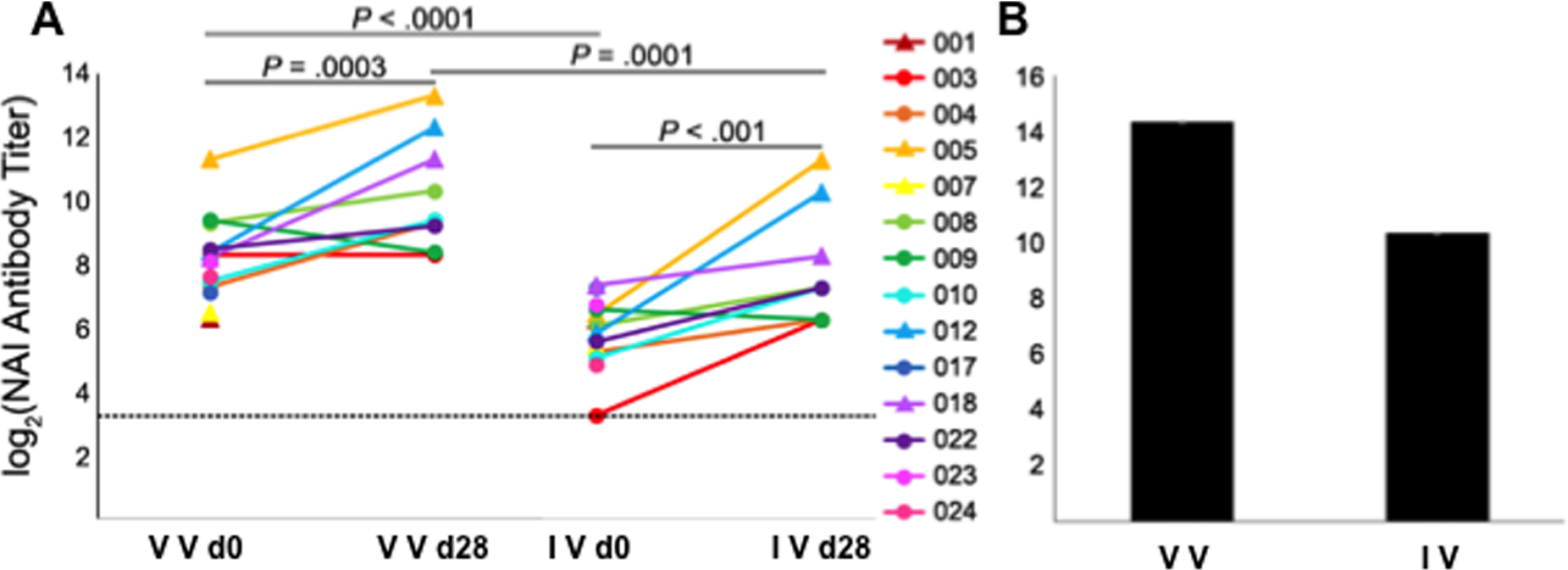
Effect of mutations present in the NA of 2015–2016 isolates on the antigenicity of the protein. ELLA assays using human (A) and ferret antisera (B). Standardized antisera from ferrets infected with the vaccine virus, and human sera collected from infected subjects at the acute (day 0) and post-acute visits 28 days later (day 28), were measured using ELLA for NAI antibodies specific for the NA protein of the vaccine strain and the virus isolated from patient 001, which encodes mutations found in the majority of 2015–2016 isolates circulating worldwide. Individual subject numbers from which the viruses were isolated are shown in the legend. Subjects whose titers are represented with circles received the 2015–2016 flu vaccine, while those represented with triangles were not vaccinated. Experiments were repeated three times, showing reproducible data. IV, isolated virus. VV, vaccine virus. The LoD is indicated by the dotted line. Experiments were repeated 3 times, showing reproducible data.

Vaccination and infection induce NA-specific protective antibodies [16,20]. It has been shown that antibodies in complex with HA sterically interfere with NA–Ab binding [68,69]. Therefore, in order to analyze NAI Ab titers, we performed enzyme-linked lectin assays (ELLAs) with H5-pseudotyped sciIAVs expressing the NA protein encoded by A/Cal/04/09 or by the circulating strain isolated from patient 001, which comprised the mutations common to the NA of pH1N1-like strains isolated worldwide during the 2015–2016 flu season, and the HA protein from a H5 virus (A/Indonesia/5/2005). Vaccine strain- and circulating strain-specific NAI Ab titers were measured using patient sera collected at day 0 and 28 days later, as well as serum from ferrets infected with A/Cal/07/09 virus (Fig 8).

In patient sera, NAI Ab titers specific to the vaccine strain and the circulating strain significantly (*P*<0.001) increased at day 28 compared to day 0 (Fig 8A), strongly suggesting that natural infection induces production of NA-specific antibodies capable of inhibiting NA sialidase activity. NAI Ab titers in human sera specific to the vaccine strain were also significantly higher (with a 4-fold average difference) than NAI Ab titers specific to the circulating strain at day 0 and at day 28 in all 14 patients analyzed (*P*<0.0001). Furthermore, an 8-fold difference in strain-specific Ab titers was observed using ferret serum (Fig 8B). These results indicate that at least one of the mutations present in the NA protein of circulating strains contribute to the evasion of NA-Ab binding.

## Discussion

To analyze whether antigenic variation could account for the low effectiveness (around 40%) of the flu vaccine during the 2015–2016 season (https://www.cdc.gov/flu/about/season/flu-season-2015-2016.htm), we characterized the HA and NA proteins of pH1N1-like IAVs and investigated the antigenic drift of strains circulating during the 2015–2016 season. Using samples collected in Rochester, NY, we found 18 and 14 mutations in the HA (Table 1 and Fig 1A and 1B) and NA (Table 2 and Fig 1C and 1D) proteins, respectively, which were representative of those found in the HA or NA of viruses currently circulating worldwide (S2 and S3 Tables, respectively). These mutations were gradually incorporated by the pH1N1-like virus population over the past seven seasons (Figs 3 and 4), and remained once incorporated (Fig 5). Additionally, these mutations are present in the HA and NA proteins of the majority of pH1N1-like strains isolated during the 2016–2017 season (S4 Table), further suggesting the stability of these mutations.

dN/dS values >1 represent amino acid positions under positive selective pressure, whereas dN/dS values <1 represent amino acid positions under negative selective pressure [41,55,57,58]. Twelve out of 18 and seven out of 14 mutations in the HA and NA proteins found in viruses circulating during the 2015–2016 season, respectively, were at codons under positive selective pressure, suggesting that these are adaptive mutations (Fig 2). Notably, not every mutation in either the HA or the NA was positively selected. Moreover, not all of the mutations in antigenic sites were subject to diversifying (poisitve) selection. That there are stable mutations present in the entire virus population located at sites subject to purifying selection could indicate that either only the substitution of certain amino acids can be tolerated at that location (due to protein functionality), or that the mutation is only beneficial under certain circumstances (i.e. necessary compensatory mutations and immune pressure).

Surprisingly, two of the mutations in the HA protein (positions 179 and 468) were predicted most likely to mutate in future pH1N1-like strains in our previous work forecasting the trajectory of pH1N1 evolution [58]. While the amino acids at the other 14 sites predicted to mutate have not changed, three of the mutations in the HA of 2015–2016 isolates (positions 180, 200, and 202) were located in the same epitopes (Sa and Sb) as three of the 14 predicted sites. While potentially useful for determining likely sites of mutations and epitopes important for protective immunity in humans, the comparison of predicted to observed sites of mutations in the 2015–2016 pH1N1-like isolates serves as a reminder that yet undefined factors contribute to selective pressures on viral proteins, and of the unpredictability of influenza viruses.

Traditionally, antigenic drift has been measured primarily with HAI assays using antisera from ferrets recovering from infection with vaccine strains [70]. However, it has been shown that after influenza virus exposure, human and ferret antisera contain antibodies that differ in the antigenic sites recognized [13,71]. In addition, immune variability exists also among different subjects due to different exposure/vaccination histories, and to the notion that the first antigenic variant encountered early in life conditions the immunity induced after infection or vaccination with seasonal strains (concept known as OAS, reviewed in [64]). To account for differences between ferret and human antisera and assess potential protective antibodies present in the human population in a more relevant manner, we have measured antigenic differences using both human sera and ferret antisera (Fig 6). In the HAI and MN assays performed using human sera, Ab titers to the circulating strains were lower than titers to the vaccine strain in some subjects (Fig 6A and 6C). Furthermore, in an mPlex HA assay, the binding of human sera to the HA protein from A/Michigan/45/2015 strain (identical in amino acid sequence to the HA from the virus isolated from patient 022) and to the vaccine strain HA proteins was not identical (Fig 7), suggesting that the mutations selected in the currently circulating viruses contribute to limited antigenic differences between the two strains. In fact, it was shown that a particular mutation (K180Q), present in the HA of isolates from the 2014–15 season as well as our 2015–16 isolates, facilitates virus escape from immune responses; however, it has no significant effect on the antigenicity of the protein measured using ferret sera [23,72]. These data indicate that the currently circulating pH1N1-like strains are not antigenically identical to the vaccine strain and reinforces the idea that using ferret sera could be misleading in some instances due to differences in the Ab repertoire among humans and ferrets [13,71].

The WHO has announced that the H1N1 strain component of the flu vaccine will be updated this coming season (2017–2018) from A/Cal/07/09 to a more recently isolated pH1N1-like strain, A/Michigan/45/2015 [73]. Remarkably, the HA and NA proteins encoded by this strain contain all of the mutations found in the majority of 2015–16 isolates described above, reinforcing the relevance of this work. It is important to note that for these assays, we used A/Cal/04/09 as the reference strain.

The majority of protective antibodies induced by vaccination or infection with influenza virus are specific to the HA protein [61,74]. As a result, most studies focus mainly on the antigenicity of the HA protein. However, protective antibodies specific to the NA protein are also elicited in animal models and humans [16,20,75,76], and antigenic drift in the NA has been reported to occur [43,44]. There were 14 mutations in the NA protein encoded by pH1N1-like viruses circulating during the 2015–16 season when compared to the vaccine strain, 4 of which were located in previously identified antigenic sites (Table 2) [51]. NAI Ab titers were lower for the circulating strain than for the vaccine strain, strongly suggesting that there is antigenic variability in the NA protein expressed by currently circulating pH1N1-like viruses–a factor which could be decreasing the efficacy of the vaccine (Fig 8). These antigenic differences could be due to the mutations in defined Ab binding sites (N200S, N248D, N369K, and K432E) [51]. Alternatively, it is very likely that because the antigenicity of the NA protein is not as well characterized, the other mutations may also affect Ab binding.

It has been shown that glycosylation on the HA head can modulate HA–receptor binding affinity [77,78], enabling some influenza viruses to escape Ab-mediated neutralization [79]. It has also been suggested that modulation of the HA/NA balance [80,81] in the event of lower HA–receptor binding affinity can occur through acquisition of compensatory mutations that decrease NA activity [82]. Interestingly, mutation S179N, found in the HA protein of viruses circulating during the 2015–16 season, is located on top of the HA head in the Sa antigenic site and incorporates an additional N-linked glycosylation site. In addition, the mutations N44S and N386K respectively incorporate and abrogate N-linked glycosylation sites in the NA. If glycosylated, the additional glycan at these sites on the HA and NA proteins could contribute to the evasion of the Ab-mediated immune response [83].

The HA stalk domain is considered relatively conserved, however genetic and antigenic differences can be detected [59,84]. Interestingly, 7 out of 18 mutations found in the pH1N1-like virus HA protein, were localized to the stalk domain. During the pandemic phase, mutations primarily accumulated in the polymerase genes and in the HA stalk domain [59]. Interestingly, mutations in the HA stalk domain are associated with protein stability and virus transmission [59], and could be influenced by immune pressure. Recently, we showed that passaging A/Cal/04/09 in the presence of immune sera or mAb specific for the stalk results in antigenic mutations [84]. Interestingly, we observed some mutations were similar or in adjacent positions in the circulating as well as the experimentally derived mutants. While not at exactly the same position, the E391K mutation in the circulating viruses is close to the A388V mutation we and other described after growing the virus in the presence of 6F12 mAb [84,85], and may be part of the same epitope. Similarly, there was a V466I mutation in the in vitro derived antigenic mutants [84], which is proximal to the S468N and N472T mutations found in currently circulating strains. Together these observations are consistent with the idea that some of the mutations we observed in the HA stalk of the circulating viruses may be in antigenic sites under immune pressure. Boosting of stalk-specific antibodies by pH1N1-like vaccination or infection, as shown in previous studies [86,87] and in our mPlex HA assays using human sera after infection (Fig 7), may increase immune pressure on pH1N1-like viruses. While the effect of the mutations selected in the HA stalk region on Ab binding remains to be determined, at least two of the mutations found (positions 338 and 391) are close to the fusion domain (Fig 1A), likely modulating Ab-mediated inhibition of membrane fusion and subsequent virus neutralization [14]. Interestingly, an amino acid substitution at position 391 in H3N2 viruses led to decreased neutralization mediated by stalk-binding antibodies [88].

In conclusion, we have shown data suggesting that the mutations present in the HA and NA proteins of 2015–2016 season isolates in Rochester (NY) have accumulated in a non-random, directional manner. The HA of pH1N1-like viruses showed limited but measurable antigenic differences to the vaccine strain. In addition, there were demonstrable antigenic differences between the NA of the pH1N1-like viruses and the vaccine strain. Additionally, infected subjects exhibited low HAI serum Ab titers against the vaccine strain, despite being vaccinated. This suggests that poor responses to the H1N1 component of the vaccine as well as antigenic differences in the HA and NA proteins of the circulating pH1N1-like viruses could be contributing to risk of infection even after vaccination. These results are yet another reminder of why we must continue surveillance efforts to better understanding the different mechanisms employed by influenza viruses in evading the Ab-mediated immune response.

## Supporting information

S1 Fig Sequence-based analysis of the HA protein used in the mPlex HA assay. (A) Sequence-based analysis of the HA subtypes. (B) Sequence-based analysis of the H1 HA proteins. Sequences are colored by subtype in A.
(TIFF)

S1 Table Patient demographics. Age, gender, weight, and vaccination history corresponding to each H1N1-positive patient (by accession number) enrolled in the acute influenza surveillance program at the University of Rochester Medical Center, NY.
(TIFF)

S2 Table Mutations in the HA proteins of pH1N1-like viruses isolated worldwide during the 2015–2016 season. Underneath in parentheses, the percentage of total strains from the respective region whose HA encoded the mutation. The total number of HA sequences from each region included in the analysis is indicated in parentheses next to the region name. Blue, yellow and pink boxes indicate amino acid residues included in Sa, Sb and Ca1 antigenic sites, respectively.
(TIFF)

S3 Table Mutations in the HA proteins of pH1N1-like viruses isolated worldwide during the 2015–2016 season. Underneath in parentheses, the percentage of total strains from the respective region whose NA encoded the mutation. The total number of NA sequences from each region included in the analysis is indicated in parentheses next to the region name. Yellow boxes indicate amino acid residues included in antigenic sites.
(TIFF)

S4 Table **Percentage of pH1N1-like strains isolated during the 2016–17 flu season whose HA (A) and NA (B) encoded the same mutations found in the HA and NA of 2015–16 isolates.** Mutations within previously defined antigenic sites are shaded gray.
(TIFF)
