# Supplementary figures and images for: Antigenicity of the 2015–2016 seasonal H1N1 human influenza virus HA and NA proteins
Source: PLoS One. 2017 Nov 16;12(11):e0188267. doi: 10.1371/journal.pone.0188267 (PMC5690631; doi:10.1371/journal.pone.0188267)

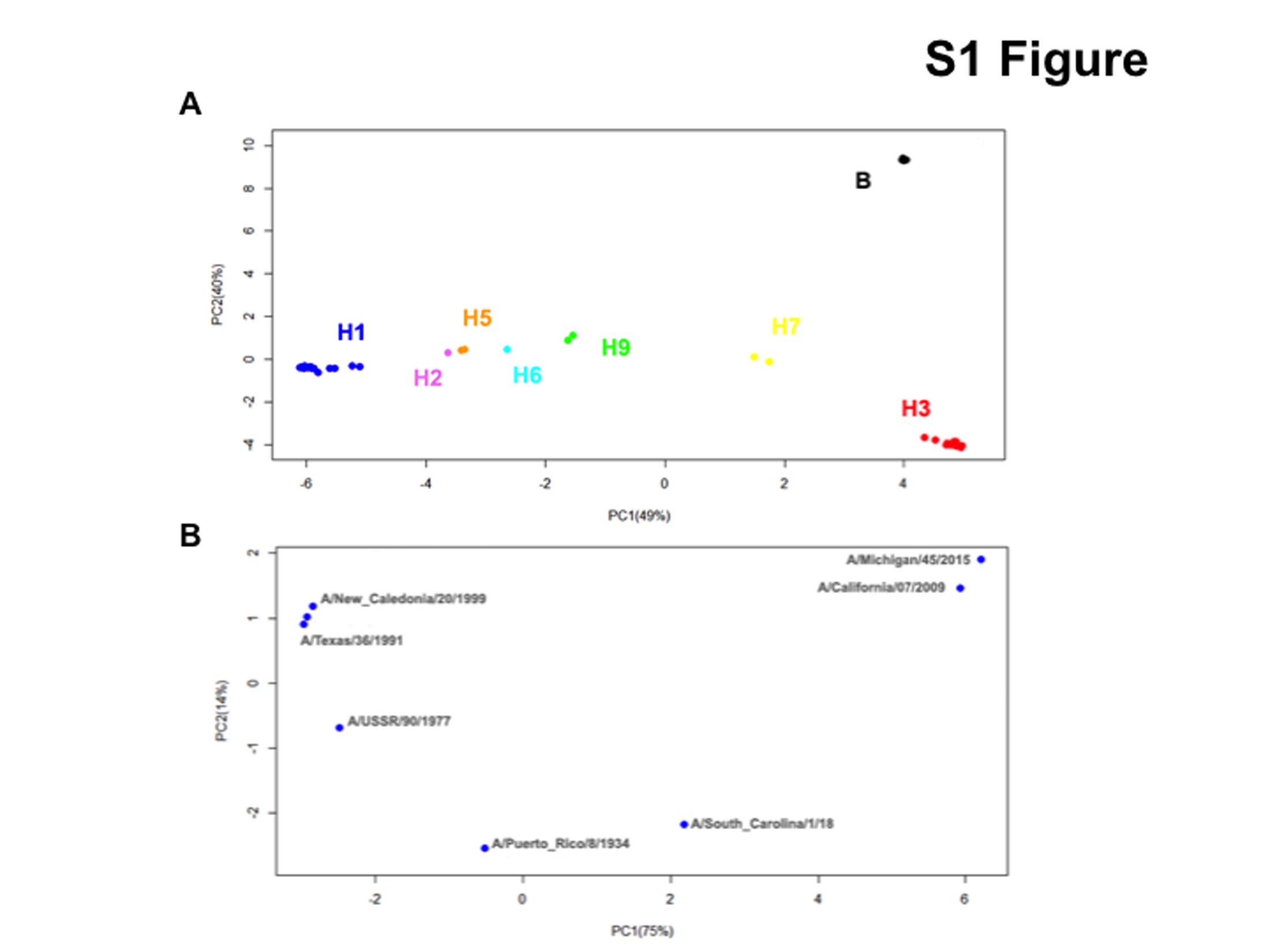

Supplement: S1 Fig — (A) Sequence-based analysis of the HA subtypes. (B) Sequence-based analysis of the H1 HA proteins. Sequences are colored by subtype in A. (TIFF) [file pone.0188267.s001.tiff]

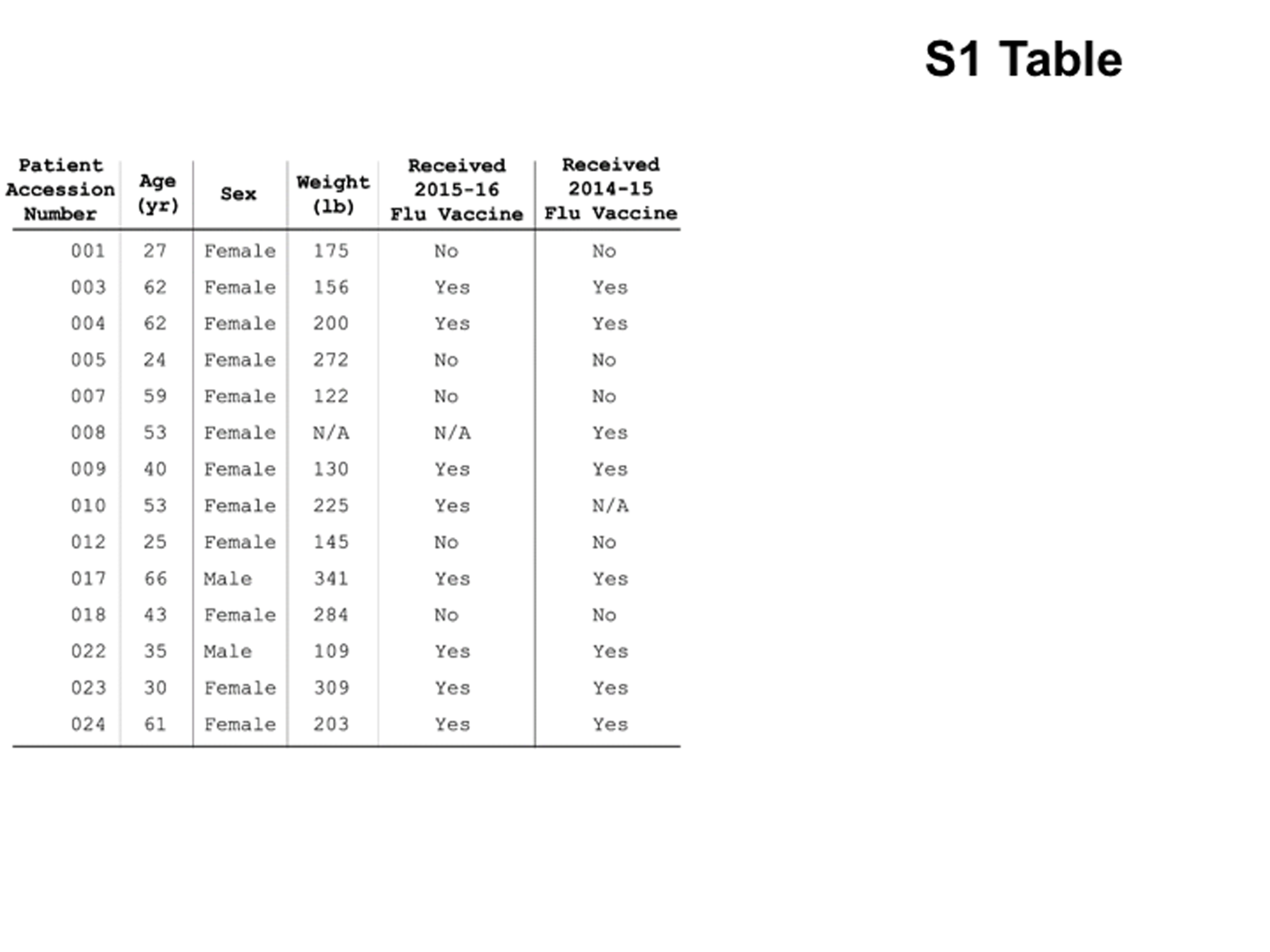

Supplement: S1 Table — Age, gender, weight, and vaccination history corresponding to each H1N1-positive patient (by accession number) enrolled in the acute influenza surveillance program at the University of Rochester Medical Center, NY. (TIFF) [file pone.0188267.s002.tiff]

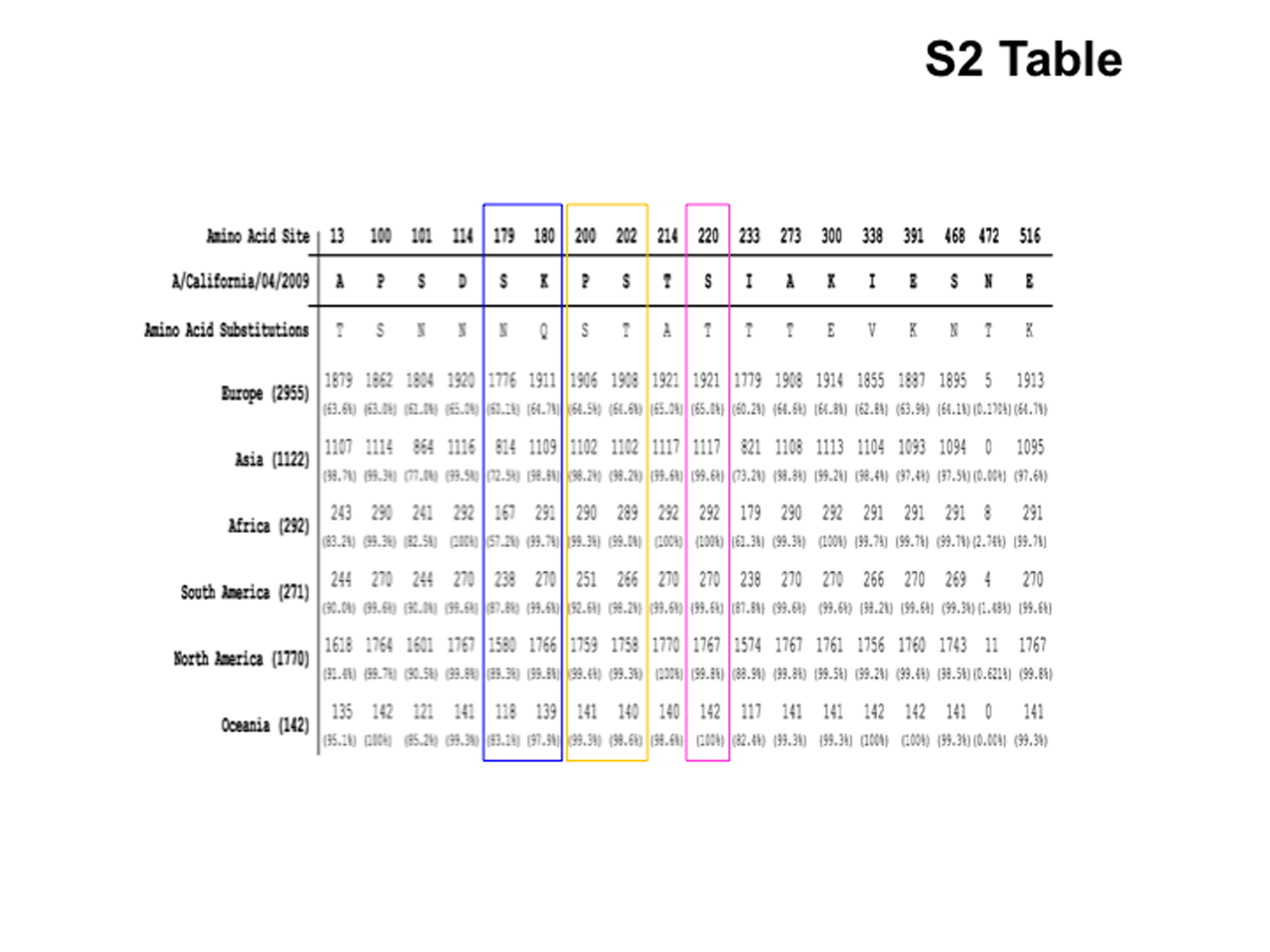

Supplement: S2 Table — Underneath in parentheses, the percentage of total strains from the respective region whose HA encoded the mutation. The total number of HA sequences from each region included in the analysis is indicated in parentheses next to the region name. Blue, yellow and pink boxes indicate amino acid residues included in Sa, Sb and Ca1 antigenic sites, respectively. (TIFF) [file pone.0188267.s003.tiff]

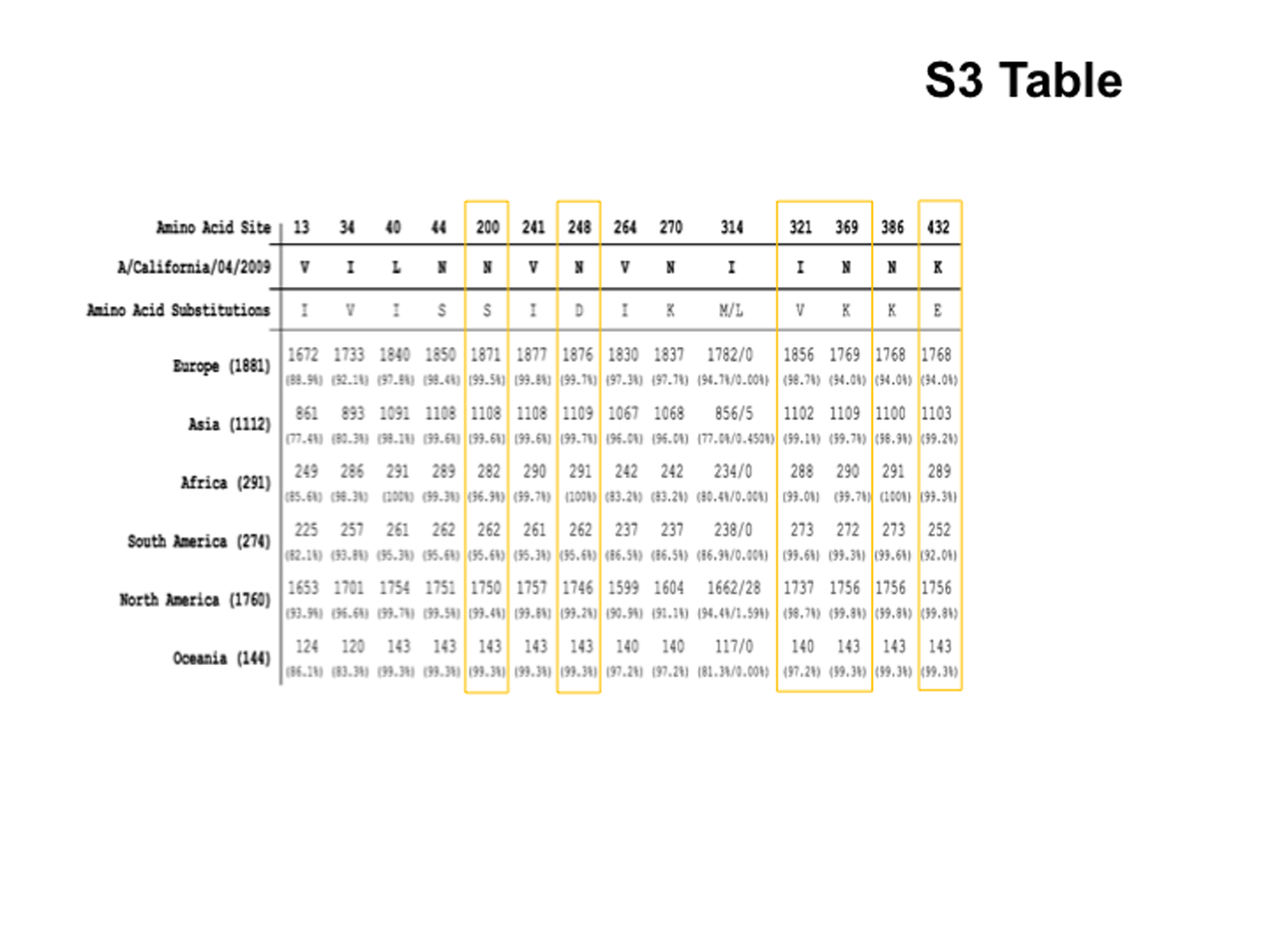

Supplement: S3 Table — Underneath in parentheses, the percentage of total strains from the respective region whose NA encoded the mutation. The total number of NA sequences from each region included in the analysis is indicated in parentheses next to the region name. Yellow boxes indicate amino acid residues included in antigenic sites. (TIFF) [file pone.0188267.s004.tiff]

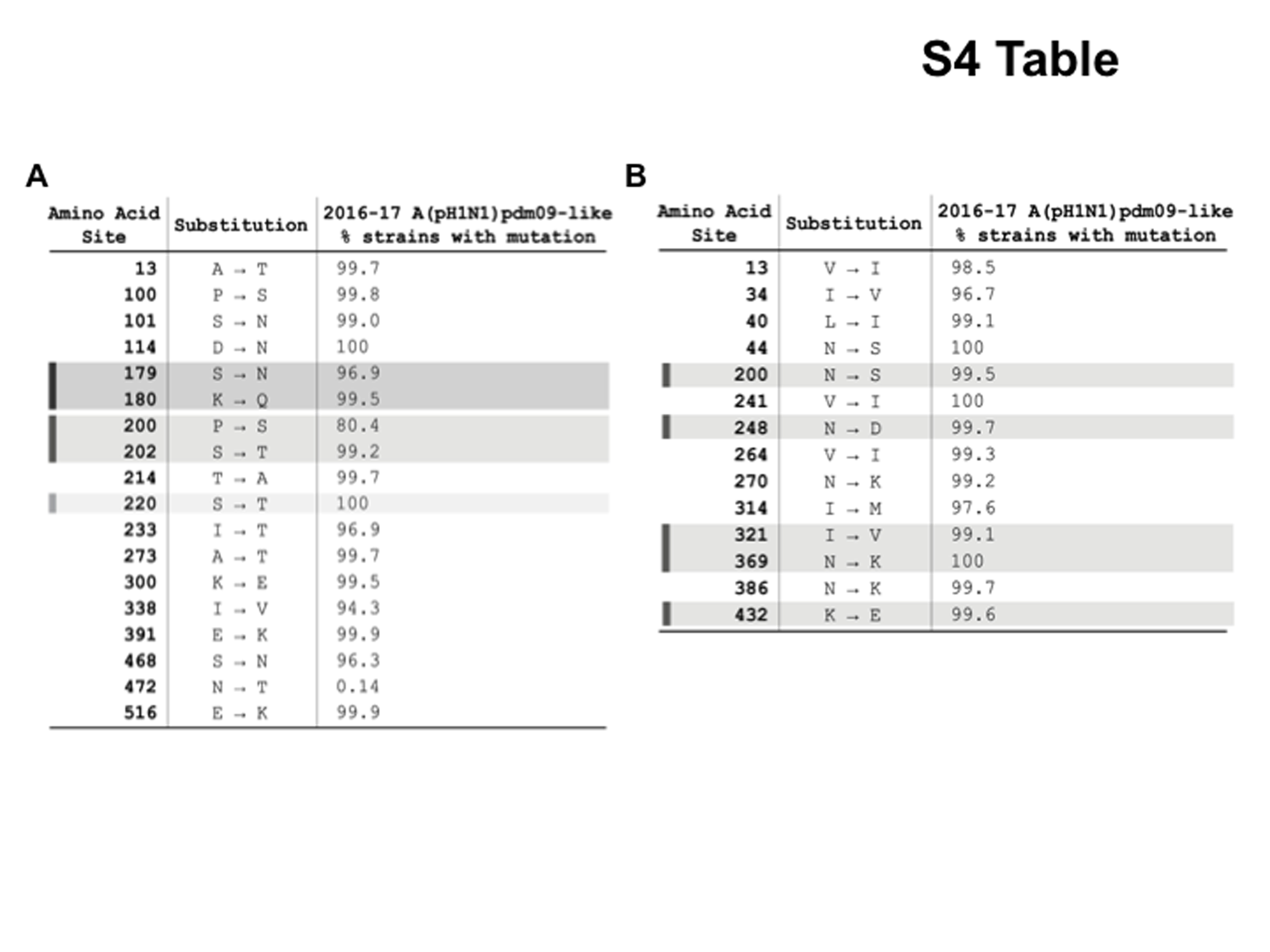

Supplement: S4 Table — Percentage of pH1N1-like strains isolated during the 2016–17 flu season whose HA (A) and NA (B) encoded the same mutations found in the HA and NA of 2015–16 isolates. Mutations within previously defined antigenic sites are shaded gray. (TIFF) [file pone.0188267.s005.tiff]
